# SARS-CoV-2 transmission across age groups in France and implications for control

**DOI:** 10.1038/s41467-021-27163-1

**Published:** 2021-11-25

**Authors:** Cécile Tran Kiem, Paolo Bosetti, Juliette Paireau, Pascal Crépey, Henrik Salje, Noémie Lefrancq, Arnaud Fontanet, Daniel Benamouzig, Pierre-Yves Boëlle, Jean-Claude Desenclos, Lulla Opatowski, Simon Cauchemez

**Affiliations:** 1 Institut Pasteur, Université de Paris, Mathematical Modelling of Infectious Diseases Unit, CNRS UMR 2000, Paris, France; 2 Collège Doctoral, Sorbonne Université, Paris, France; 3 Santé publique France, French National Public Health Agency, Saint-Maurice, France; 4 Univ Rennes, EHESP, REPERES (Recherche en Pharmaco-Epidémiologie et Recours aux Soins), EA 7449 Rennes, France; 5 Department of Genetics, University of Cambridge, Cambridge, UK; 6 Institut Pasteur, Université de Paris, Emerging Diseases Epidemiology Unit, Paris, France; 7 Conservatoire National des Arts et Métiers, PACRI Unit, Paris, France; 8 Sciences Po - Centre de sociologie des organisations and Chaire santé - CNRS, Paris, France; 9 Sorbonne Université, INSERM, Institut Pierre Louis d’Epidémiologie et de Santé Publique, Paris, France; 10 Université Paris-Saclay, UVSQ, Inserm, CESP, Anti-infective evasion and pharmacoepidemiology team, Montigny-Le-Bretonneux, Gif-sur-Yvette, France; 11 Institut Pasteur, Université de Paris, Epidemiology and Modelling of Antibiotic Evasion (EMAE), Paris, France

**Keywords:** Computational models, Statistical methods, SARS-CoV-2, Epidemiology

## Abstract

The shielding of older individuals has been proposed to limit COVID-19 hospitalizations while relaxing general social distancing in the absence of vaccines. Evaluating such approaches requires a deep understanding of transmission dynamics across ages. Here, we use detailed age-specific case and hospitalization data to model the rebound in the French epidemic in summer 2020, characterize age-specific transmission dynamics and critically evaluate different age-targeted intervention measures in the absence of vaccines. We find that while the rebound started in young adults, it reached individuals aged ≥80 y.o. after 4 weeks, despite substantial contact reductions, indicating substantial transmission flows across ages. We derive the contribution of each age group to transmission. While shielding older individuals reduces mortality, it is insufficient to allow major relaxations of social distancing. When the epidemic remains manageable (R close to 1), targeting those most contributing to transmission is better than shielding at-risk individuals. Pandemic control requires an effort from all age groups.

## Abstract (web summary)

In this study, Tran Kiem et al. examine the contribution of different age groups to COVID-19 transmission. Using data from the French epidemic in summer 2020, they report that while individuals aged 80 years and older are more at risk, pandemic control in the absence of vaccines required measures targeted at all age groups.

## Introduction

To mitigate the impact of COVID-19 during the first year of the pandemic, many countries implemented drastic social distancing measures that proved effective at reducing the stress on the healthcare system^1,2^ but were associated with major social and economic costs because they required an effort from all. Since infections leading to hospitalization and death were concentrated in elderly people and people with comorbidities, some argued that strategies that shield at-risk individuals from infection (for example, by isolating them) could be used to maintain hospitalizations at low levels while relaxing costly social distancing measures that affect the rest of society^3,4^, which has raised substantial debates^5–7^. These arguments resonate with decades-old debates on the relative contribution to disease control of strategies that target at-risk individuals versus disease transmitters^8–13^.

The massive roll-out of safe and effective vaccines^14–16^ should ensure that countries no longer need to resort to drastic social distancing measures such as lockdowns to control COVID-19 epidemics. Nonetheless, it remains important to determine whether, in the absence of vaccines, strategies shielding at-risk individuals may allow the relaxation of social distancing measures since: (i) COVID-19 vaccine coverage remains low in many countries and (ii) shielding strategies may be considered at the start of future emergences when no vaccines are available yet. Such evaluation requires a detailed understanding of the dynamics of transmission of SARS-CoV-2 across age groups. We perform such assessment by analyzing the epidemic rebound that occurred in France in the summer–autumn 2020. In France, the nationwide lockdown implemented in spring 2020^1^ was followed by the progressive relaxation of social distancing measures, the scaling up of a strategy based on testing, contact tracing and case isolation and the general use of face masks. However, this did not impede a large second wave in the autumn and a new lockdown in November 2020.

Here, we build a modeling framework to reconstruct the complex patterns of spread of SARS-CoV-2 across age groups along with the dynamics of infections and hospitalizations, from the detailed analysis of age-stratified case (*N* = 368,906) and hospitalization (*N* = 16,548) data from all 13 regions of Metropolitan France, between 15 June and 28 September 2020. We fit our model to age-stratified hospital admissions and positivity rates among symptomatic individuals that received a RT-PCR test result (labeled symptomatic individuals in the rest of the text). Based on these dynamics, it is possible to quantify the contribution of each age group to transmission. This characterization can then be used to critically evaluate different age-targeted intervention measures implemented in the absence of vaccines. We only consider interventions targeting members of the general population (i.e., we do not assess measures targeting specific settings such as elderly homes, hospitals, or prisons). We first detail the results for Auvergne-Rhône-Alpes (8 million inhabitants), which was one of the first regions to experience an epidemic rebound (Supplementary Fig. 1); and then present the results for all 13 regions in metropolitan France.

## Results

### Epidemic dynamics across age groups

**Fig. 1:**
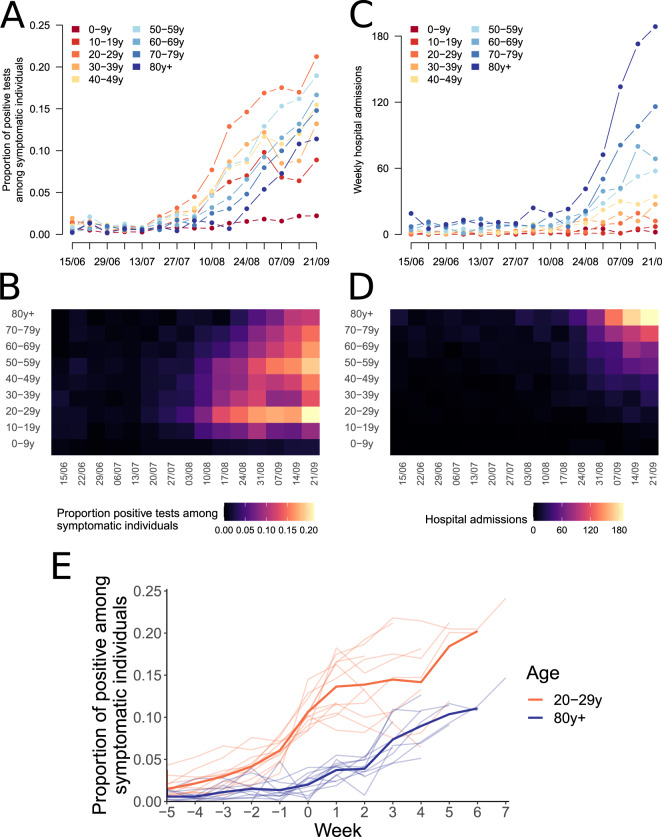
Dynamics of the epidemic rebound by age group. **A**, **B** Weekly proportion of positive tests amongst symptomatic individuals being tested, and **C**, **D** weekly number of hospital admissions, by age group in Auvergne-Rhône-Alpes region. **E** Proportion of positive tests among symptomatic individuals in individuals aged 20–29 yr and older than 80 yr. In panel (**E**), the light lines represent the trends in the 13 metropolitan French regions. The wider lines indicate the mean proportion of positive among symptomatic across regions. Week 0 corresponds to the first week when the proportion of positive tests among symptomatic individuals aged 20–29 yr reaches 8%.

In the Auvergne-Rhône-Alpes region, the proportion of positive tests among symptomatic individuals aged 20–29 yr increased from 3.2% to 12.9% between 27 July 2020 and 17 August 2020 (Fig. 1A). This increase was quickly followed by a rise in positivity rates (Fig. 1A, B) and hospital admissions in other age groups (Fig. 1C, D). For example, on the week of 14 September 2020, 10.8% of symptomatic individuals aged ≥80 yr were positive (compared to 0.7% on the week of 17 August 2020) and there were 169 hospital admissions of patients in that age group (compared to 23 on the week of 17 August 2020). These trends were observed across all metropolitan French regions, with a mean lag of 4 weeks between the increase in the proportion of positive tests among symptomatic individuals aged 20–29 yr and those older than 80 yr (Fig. 1E). This indicates substantial porosity of transmission between age groups.

### Estimates of the contribution of different age groups to transmission

**Fig. 2:**
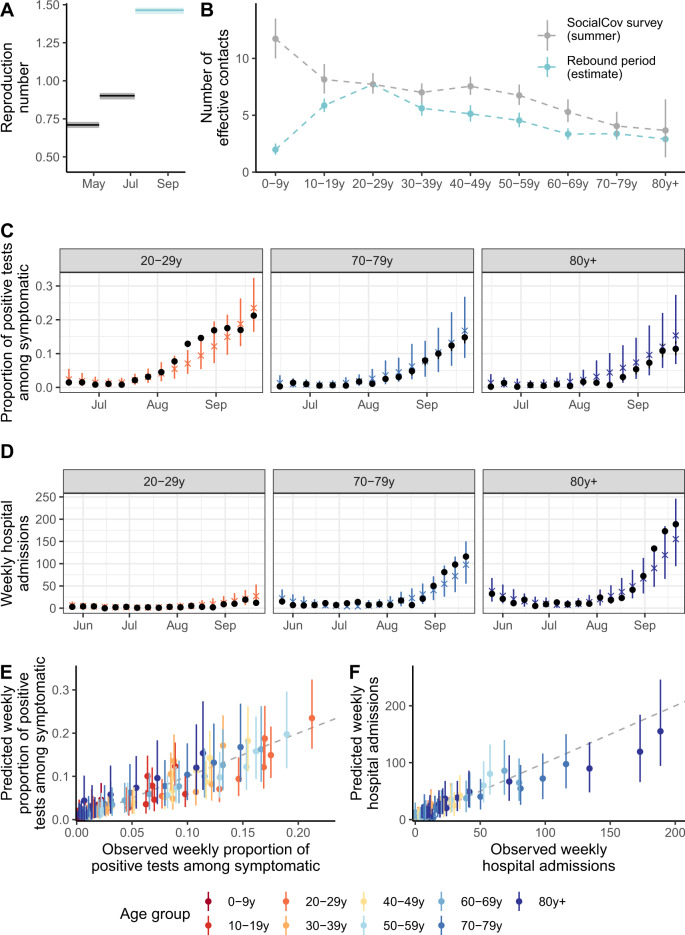
Model predictions for Auvergne-Rhône-Alpes region. **A** Intervention reproduction number estimates during the epidemic. **B** Effective number of contacts estimated for each age group during the rebound period (9 July–27 September). **C** Predicted and observed weekly proportion of positive tests amongst symptomatic individuals being tested aged 20–29 yr, 70–79 yr, and 80 yr+. **D** Predicted and observed weekly number of hospitalizations of individuals aged 20–29 yr, 70–79 yr, and 80 yr+. **E** Predicted and observed weekly proportion of positive tests among symptomatic individuals being tested. **F** Predicted and observed weekly hospital admissions. In panel (**A**), the shaded areas correspond to 95% credible intervals obtained from the posterior distribution. The points and vertical segments for the blue curve in panel (**B**) correspond to the means and 95% credible intervals obtained from the posterior distribution (Markov Chain Monte Carlo (MCMC) chain of 100,000 iterations removing 5000 iterations of burn-in). The points and vertical segments for the gray curve correspond to the observed mean and to 95% bootstrap confidence intervals (10,000 bootstrap samples). The black points in panels (**C**, **D**) indicate the data. The colored crosses and vertical segments in panels (**C**, **D**) indicate the means and 95% credible intervals obtained from 500 simulations from the posterior distribution. In panels (**E**, **F**), each point corresponds to a specific week and age group. The colored points and vertical segments in panels (**E**, **F**) indicate the means and 95% credible intervals obtained from 500 simulations from the posterior distribution.

To quantify the impact of interventions over time, it is important to note that effective reproduction numbers naturally decline as the proportion of susceptible individuals declines, even if transmission rates remain the same. Here, we introduce the intervention reproduction number *R*_*i*_ as the average number of infections resulting from a single index case under a set of interventions if the population was completely susceptible. Fitting our model to these data, we estimate that, in Auvergne-Rhône-Alpes, *R*_*i*_ increased from 0.71 (95% credible interval: 0.69–0.73) during the lockdown to 0.90 (95% CrI: 0.88–0.93) between 11 May and 8 July and to 1.46 (95% CrI: 1.44–1.49) from 9 July to 28 September 2020 (Fig. 2A and Supplementary Table 1).

We define daily effective contacts as model predicted daily contacts in the estimated mixing matrix rescaled so that the number of daily effective contacts in the 20–29 years old (y.o.) is 7.7, as observed in the SocialCov survey (Supplementary Fig. 2). We estimate that the number of effective contacts in the rebound period starting on 9 July was highest in individuals aged 20–29 yr (Fig. 2B and Supplementary Figs. 2 and 3). As a comparison, the number of effective contacts in those aged 10–19 yr, 50–59 yr, and ≥80 yr was, respectively, 5.9 (5.3–6.5), 4.5 (3.9–5.2), and 2.9 (2.4–3.4), corresponding to 0.76 (0.69–0.84), 0.59 (0.51–0.68), and 0.38 (0.31–0.45) times the number of effective contacts in individuals aged 20–29 yr. These estimates are consistent with the number of daily contacts measured in different age groups by the online survey SocialCov (30 July–27 September 2020) (see Supplementary information)^17^, but for two key differences (Fig. 2B). First, we estimated that the number of effective contacts for transmission in children aged 0–9 yr was substantially lower than the reported number of contacts in the survey. This reflects the limited contribution of younger children (0–9 y.o.) to SARS-CoV-2 transmission during this time period and is consistent with either a lower susceptibility to SARS-CoV-2 infection or a reduced infectivity compared to older individuals^18–21^. Second, the contribution to transmission of all other age groups relative to those aged 20–29 yr is between 17% and 37% lower than what might be expected from the contact survey. Again, this might be explained by reduced risks of transmission given contact compared to 20–29 y.o., for example, thanks to better compliance with the use of masks or physical distancing. These differences highlight the distinction between raw contacts measured from contact surveys and effective contacts that we estimate and that capture different risks of transmission given contact. Our estimated mixing patterns can reproduce the observed rises in positivity rates (Fig. 2C, E and Supplementary Figs. 4 and 5) and hospital admissions by age group (Fig. 2D, F and Supplementary Figs. 4 and 6).

### Impact of strategies shielding the elderly population in the absence of vaccines

We use our model to assess the potential impact of social distancing measures targeting different age groups in the absence of vaccines. We further assume that when individuals reduce their contacts, their contacts are affected homogeneously irrespective of their age.

**Fig. 3:**
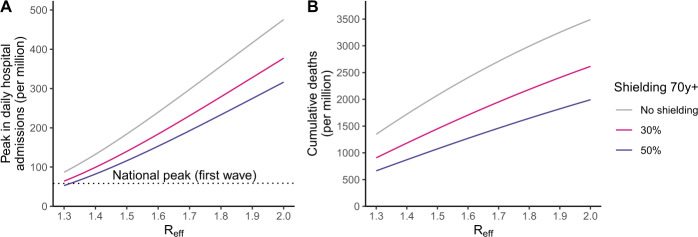
Impact of strategies shielding the elderly population. **A** Peak in hospital admissions per million, and **B** number of deaths per million as a function of the effective reproduction number *R*_eff_ assuming a reduction of 50% or 30% in effective contacts of those older than 70 yr. The number of deaths is computed from the time interventions are implemented until the end of the simulation, corresponding to the period from 28 September 2020 to 1 January 2022. The impact of reducing contacts in individuals aged 70 yr and older in counterfactual simulations was reported according to the effective reproduction number at the start of the simulation. The effective reproduction number decreased over the course of the simulation with increasing immunity.

In Auvergne-Rhône-Alpes, the effective reproduction number *R*_eff_ (i.e., the average number of individuals infected by an index case accounting for the build-up of immunity) increased from 1.3 to 1.5 during the build-up of the autumn wave^22–24^. Even though this corresponds to a 50% reduction in the transmission rate compared to a scenario with no control measures^1^, this was insufficient to avoid a surge in hospitalizations and eventually the implementation of a national lockdown on 30 October 2020. We explore whether shielding individuals aged ≥70 yr could have been sufficient to maintain the epidemic at manageable levels for hospitalizations while relaxing control measures so that the effective reproduction number would be *R*_eff_ ≥ 1.3–1.5. We deliberately consider an “extreme” scenario of shielding where the number of effective contacts of the target age group would be reduced by 50% to be similar to what was measured during the lockdown of March–May 2020^17^. Going further than this reduction seems difficult as this lockdown was already very strict. We find that in the range *R*_eff_ = 1.3–1.5, this would still result in 53–116 per million daily hospital admissions at the peak, above the national peak of March–April 2020 (56 per million) (Fig. 3A) and 664–1074 deaths per million (Fig. 3B). Further relaxing control measures up to *R*_eff_ = 1.8 would increase the peak daily number of hospitalized patients to 233 per million and the overall number of deaths to 1646 per million. Applying these reductions to individuals ≥60 y.o. would not avoid a surge of COVID-19 patients in hospitals, shall control measures be relaxed (Supplementary Fig. 7).

### Impact of strategies targeted towards different age groups

**Fig. 4:**
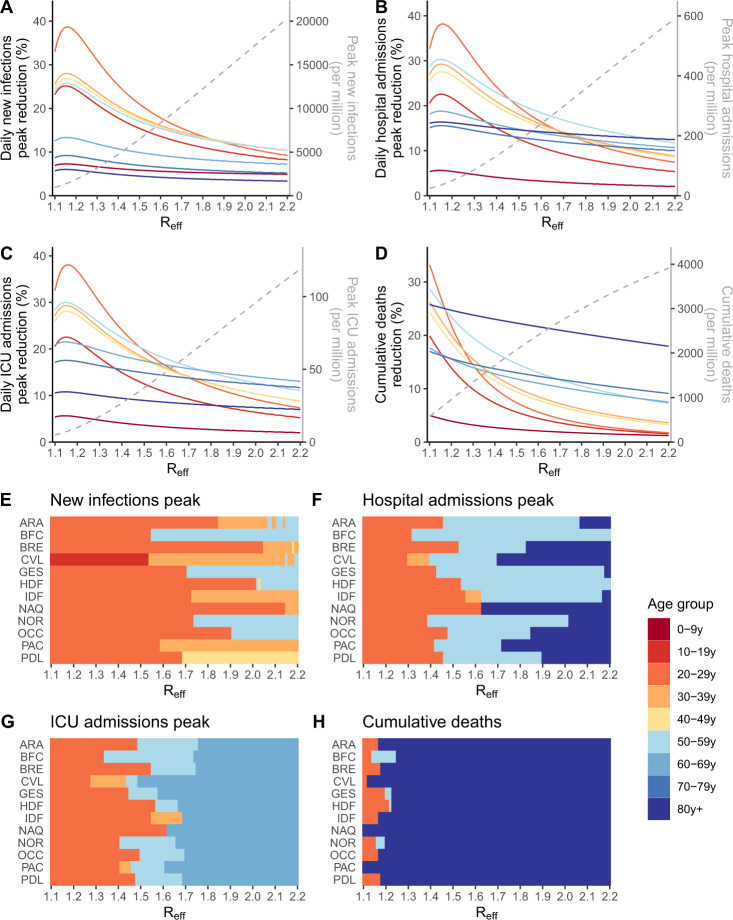
Impact of strategies targeting specific age groups. Reduction in (**A**) the peak in daily new infections, (**B**) the peak in hospital admissions, (**C**) the peak in daily ICU admissions, (**D**) the number of deaths when individuals in the target age group reduce their effective contacts by 1, as a function of the effective reproduction number *R*_eff_, in the Auvergne-Rhône-Alpes region. The gray dotted lines indicate, in the absence of additional measure, the value of the epidemiological metrics. Age groups for which a reduction of 1 contact results in the highest impact on the reduction of (**E**) the peak in daily new infections, (**F**) the peak in hospital admissions, (**G**) the peak in daily ICU admissions, and (**H**) the number of deaths as a function of the effective reproduction number *R*_eff_. In counterfactual simulations, the impact of reducing 1 effective daily contacts in each age group from the region-specific date of beginning of simulation (Table S4) to 1 January 2022 was compared for different values of the effective reproduction numbers at the beginning of the simulations, which then declined in the simulation with increasing immunity. The number of deaths is computed from the time interventions are implemented until the end of the simulation. Region’s abbreviations are detailed in supplementary text.

This suggests that shielding elderly individuals would not allow an important relaxation of social distancing measures as the effective reproduction number needs to be maintained close to 1 for the epidemic to remain manageable. This requires efforts from all age groups. In this latter context of a slowly growing epidemic characterized by *R*_eff_ close to 1, we investigate if it would be better from a public health perspective to reduce contacts of elderly individuals rather than those of other age groups. We find that, for *R*_eff_ close to 1, targeting 20–29 y.o. individuals, i.e., the age group with the largest number of effective contacts, results in the largest reduction in key epidemiological metrics. For example, considering the example of the region Auvergne-Rhône-Alpes, in a scenario where *R*_eff_ = 1.1, the peaks in new infections (Fig. 4A), hospital admissions (Fig. 4B), ICU admissions (Fig. 4C), and the number of deaths (Fig. 4D) would all drop by 33%, if all individuals aged 20–29 yr reduced their average number of effective contacts by 1 (i.e., from 7.7 contacts per day to 6.7 on average), compared to 6%, 16%, 11%, and 26%, respectively, if those aged ≥80 yr were targeted instead (from 2.9 to 1.9 contacts per day on average).

We found in the previous section that the healthcare system would be unable to cope with large values of the reproduction number even if elderly individuals were shielded. We nevertheless explore such scenarios in case the cost of control measures was judged too elevated by decision makers. As the reproduction number increases, the same efforts in terms of reductions of contacts would lead to lower impact on key epidemiological metrics; and the ordering of strategies may change towards a higher efficiency of strategies targeting those most at risk of severe outcomes. Targeting ≥80 y.o. individuals becomes the best strategy to reduce deaths when *R*_eff_ is ≥1.17 (Fig. 4D). For instance, if *R*_eff_ = 1.6, the number of deaths would drop by 22% if we removed 1 effective contact for those aged 80 yr and older; but by only 6% if we targeted those aged 20–29 yr. We find a similar pattern if the objective is to minimize the number of life-years lost and quality-adjusted life years (Supplementary Fig. 8). For large values of *R*_eff_, we obtain relatively similar reductions on peak hospital admissions irrespective of the target group among all age groups ≥20 y.o. To reduce peak ICU admission, it remains slightly less interesting to target those aged ≥80 yr since this population is less likely to be admitted in ICU. The largest reduction in the peak number of infections is always obtained targeting groups significantly contributing to transmission irrespective of the value of *R*_eff_. These conclusions remain unchanged when a larger number of effective contacts is being removed, although the impact on epidemiological metrics increases (Supplementary Figs. 9 and 10).

**Fig. 5:**
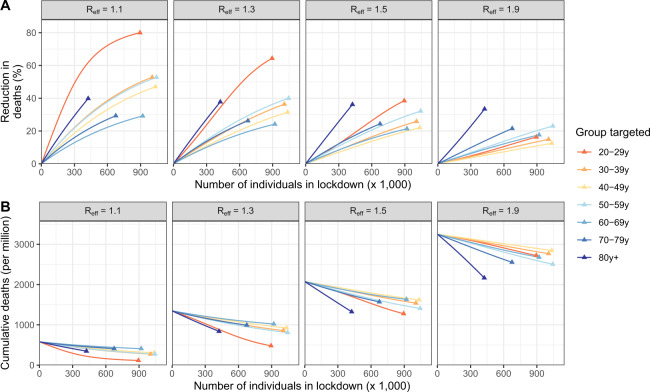
Impact of targeted strategies as a function of the equivalent number of individuals put into lockdown in the different age groups. **A** Percentage reduction in cumulative deaths, and **B** remaining cumulative deaths in the Auvergne-Rhône-Alpes region for strategies targeting different age groups. The results are presented for different values of the effective reproduction number *R*_eff_ at the beginning of the simulations, which then declined in the simulation with increasing immunity. Simulations are run for different intensities of targeting. For each targeted strategy, we compute the equivalent number of individuals that would need to be put into lockdown to reach this level. The lockdown of an entire age group corresponds to the triangle.

As the number of effective contacts differs between age groups (Fig. 2B), a reduction of 1 effective contact does not correspond to the same effort in the different age groups. For example, removing 1 effective contact per day corresponds to a 13% reduction of contacts in individuals aged 20–29 yr, but a 35% reduction in those aged ≥80 yr. Applying the same 20% reduction of effective contacts in all age groups, we find that the largest reduction in the peak of new infections, hospital admissions and ICU admissions is obtained when targeting the 20–29 y.o. regardless of the effective reproduction number value (Supplementary Fig. 11). The optimal strategy to minimize the number of deaths targets those aged ≥80 yr when *R*_eff_ ≥ 1.46 (compared to ≥1.17 for an absolute reduction of 1 contact) (Supplementary Fig. 12). To account for the fact that different age groups have different numbers of contacts and different capacities to reduce contacts, we can also compare strategies where the same number of individuals are put into lockdown in the different age groups (Supplementary Fig. 13). In this scenario, we also find that optimal strategies shift from targeting those that contribute the most to transmission for *R*_eff_ < 1.3 (Fig. 5) to targeting older individuals for larger values of *R*_eff_. However, for these larger values of *R*_eff_, the lockdown of those aged 80 yr and older would still result in a significant mortality (e.g., 2170 deaths per million for *R*_eff_ = 1.9).

### Results across regions in metropolitan France

Our model can reproduce the dynamics of test positivity in symptomatic individuals and hospitalizations across all the regions of metropolitan France (Supplementary Figs. 14–25). We also find consistent patterns regarding the numbers of effective contacts by age group across regions (Supplementary Fig. 26), with the highest values observed in individuals aged 20–29 yr. In 10 out of 12 regions of Metropolitan France, we reach similar conclusions that in situations characterized by *R*_eff_ close to 1 where the epidemic may remain manageable, it is beneficial to reduce effective contacts of those that contribute the most to transmission; while for larger values of *R*_eff_ that are likely to lead to a major crisis in hospitals, it is optimal to target those with the highest risk of severe outcome (Fig. 4E–H and Supplementary Fig. 8). The two regions where we find it is beneficial to start targeting older individuals to maximize the reduction in deaths when *R*_eff_ is low are characterized by low estimates of the number of contacts in those aged ≥80 yr (respectively, 1.55 (1.03–2.18) for Nouvelle-Aquitaine and 2.38 (1.77–3.09) for Provence-Alpes-Côte d’Azur) and are the metropolitan French regions with the highest proportion of ≥80 y.o. in their population.

### Sensitivity analyses

**Fig. 6:**
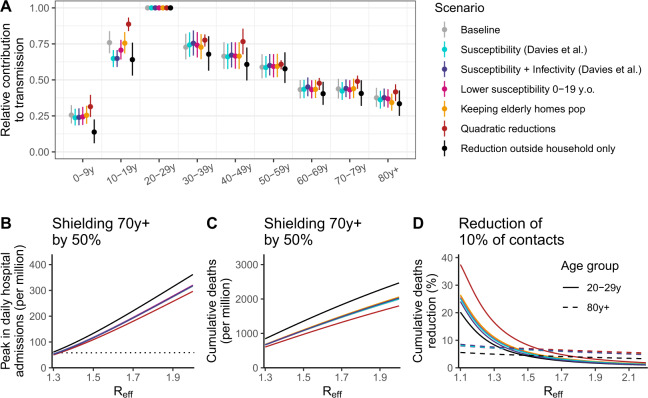
Sensitivity analyses for the Auvergne-Rhône-Alpes region. **A** Relative contribution of the different age groups to transmission compared to the 20–29 y.o. age group across a range of scenarios. **B** Peak in daily hospital admissions (per million inhabitants) assuming a reduction of 50% in contacts of those older than 70 yr across a range of scenarios as a function of the effective reproduction number *R*_eff_. **C** Number of deaths (per million inhabitants) assuming a reduction of 50% in contacts of those older than 70 yr across a range of scenarios as a function of the effective reproduction number *R*_eff_. **D** Reduction in the number of deaths (reported in percentage) as a function of the effective reproduction number *R*_eff_ for strategies targeting those aged 20–29 yr and those 80 y.o. and older. The horizontal dotted line in panel (**B**) corresponds to the peak in daily hospital admissions observed at the national level during the first pandemic wave of SARS-CoV-2. The scenarios explored are: Susceptibility (Davies et al.)—using age-specific susceptibilities^21^; Susceptibility + Infectivity (Davies et al.)—using age-specific susceptibilities and infectivities^21^; Lower susceptibility 0–19 y.o.—0–9 y.o. and 10–19 y.o. are, respectively, 50% and 25% less susceptible to SARS-CoV-2 infection than 20 y.o. and older; Keeping elderly homes pop—including the population of elderly homes in the study population; Quadratic reduction—considering quadratic reductions in contact patterns; Reduction outside household only—assuming contact patterns are only modified outside the household. In counterfactual simulations, the impact of the targeted strategies from 28 September 2020 to 1 January 2022 was compared for varying, counterfactual degrees in effective reproduction numbers at the beginning of the simulations, which then declined in the simulation with increasing immunity. In panel (**A**), the points and vertical segments correspond to the means and 95% credible intervals obtained from the posterior distribution (Markov Chain Monte Carlo (MCMC) chain of 100,000 iterations removing 5000 iterations of burn-in).

In a sensitivity analysis, we vary assumptions about the relative infectivity and susceptibility of the different age groups and the way we model the impact of interventions targeting different age groups. We find consistent results regarding the contribution of age groups to transmission (Fig. 6A and Supplementary Fig. 27). In all scenarios, individuals aged 20–29 yr contribute the most to transmission, children aged 0–9 yr have a limited contribution (between 0.14 and 0.31 times the contribution of the 20–29 y.o. across scenarios) and among those aged 20 yr and older, the contribution of the different age groups decreases with age. Across these scenarios, the magnitude of the contribution to transmission of the 10–19 y.o. is roughly similar to that of the 30–49 y.o. We find higher heterogeneity between age groups when assuming that contacts are only modified outside the household and a lower heterogeneity when considering quadratic reductions in contact patterns. Interestingly, we find similar estimates when varying assumptions regarding the infectivity and susceptibility of the different age groups, which suggests that the notion of effective contacts captures the actual contribution of the different age groups to transmission, including their varying infectivity or susceptibility. Across these scenarios, we explore the correlation between the number of contacts reported in the SocialCov contact survey and the number of contacts estimated, by adjusting our estimated effective contacts for changing assumptions regarding the infectivity and susceptibility of the different age groups. Accounting for a reduced susceptibility in those aged 0–19 yr provides the highest correlation (Supplementary Fig. 28). Exploring the impact of strategies targeting specific age groups across these sensitivity analyses, we find that the shielding of older individuals is insufficient to avoid an important surge in hospitalizations and deaths (Fig. 6B, C) and that the most efficient strategy to minimize deaths shifts from targeting those that contribute most to transmission to those most at risk of severe outcomes as *R*_eff_ increases (Fig. 6D).

## Discussion

At the start of the COVID-19 autumn wave in 2020, we observed a very consistent epidemiological pattern across the 13 regions of metropolitan France. It started with an increase of infections among young adults, which was followed up by a rise in infections in other age groups and eventually in older individuals. Similar patterns have been described in other locations^25–27^. This indicates substantial porosity of transmission across age groups. We used our model to quantify this phenomenon and evaluate non-pharmaceutical control strategies targeting different age groups. We found that even if we managed to reduce effective contacts of older individuals by 50%, this would not allow important relaxations of control measures in the absence of vaccines. In practice, it is unclear whether it would be possible to achieve such reductions for this age group since (i) older individuals already behave very carefully with a number of effective contacts that is 2–5 times lower than that of those aged 20–29 yr and (ii) they are often dependent persons with a minimum number of contacts required for their basic daily activities. In all instances, our results indicate that to avoid a major crisis in hospitals, in the absence of vaccines, it is essential to maintain transmission rates at relatively low levels (with *R*_eff_ close to 1) which requires an effort from all. For this parameter regime where *R*_eff_ is close to 1, reducing contacts in younger age groups who contribute more to transmission would have a larger impact on key epidemiological indicators than targeting at-risk individuals.

Besides, strategies based on shielding a single part of the population, like the elderly, may raise serious ethical and social concerns. Such strategies can easily fuel societal controversies undermining social cohesion (“age-itation”), often viewed as a key asset in the management of the epidemic^28,29^. Differentiated strategies might also modify the compliance of certain groups to other measures, which could reduce their impact. From a broader social perspective, the focus on the elderly would also represent a breach in values of solidarity between citizens and generations, which is considered as a cement of the welfare state in countries like France. The isolation of the elderly would erode social ties and weaken their situation, with strong concerns on ethical principles such as autonomy and benevolence^30^. From a wider political perspective, such strategies would also represent a shift in the legitimacy of the State to intervene to control the epidemic: by promoting self-protection strategies rather than collective measures, governments will weaken their own capacity to intervene, leaving ground to more individualistic strategies.

We critically evaluated measures targeting members of the general population of different age groups without assessing measures targeting specific settings such as elderly homes, hospitals, or prisons, where transmission dynamics are expected to be different^31^. In France, like in a number of other countries, elderly home residents were strongly impacted by the pandemic, representing more than 40% of deaths until February 2021. Shielding elderly home residents was therefore rightly considered a priority to mitigate pandemic impact. Here, we investigated whether, in addition to epidemic control in elderly homes, shielding of individuals aged 70 yr and older that do not live in elderly homes (about 93% of the age group^32^) might allow important relaxation of control measures in the absence of vaccines. This was done by excluding elderly home residents from our assessment, therefore considering a best case scenario where these individuals are completely protected from infection. The impact of shielding would be strengthened if the target group (70 y.o. and older) was to be extended to those aged 60 yr or to younger individuals with comorbidities. However, a lot of individuals aged 60–70 yr have not retired yet raising feasibility issues; and age has been found to be the primary driver of severity^33–37^ so that this would be unlikely to change our key conclusions. We found similar patterns running a sensitivity analysis including the population of elderly homes in our study population.

Fortunately, the advent of safe and effective vaccines has greatly expanded our toolkit for epidemic control beyond non-pharmaceutical measures. The progressive roll-out of vaccines has reduced the COVID-19 burden by protecting elderly individuals from severe outcomes and by reducing viral circulation^38,39^. Interestingly, we found similarities between the question of vaccine doses’ prioritization towards different age groups and that of contact reduction explored here. Modeling studies have highlighted that, if vaccines are highly effective against infection, vaccinating young adults could be the best way to minimize mortality in a low-transmission setting. However, as transmission increases, the optimal strategy switches to vaccinating older individuals^38–40^. This is consistent with our assessment of how optimal target groups may change with *R*_eff_.

Case data can be difficult to interpret as they are sensitive to (i) changes in testing capacities and policies and (ii) age-specific characteristics (e.g., propensity to get tested or probability to develop symptoms). In this study, we propose a modeling framework relying on the analysis of the dynamics of the proportion of positive tests among individuals reporting symptoms upon getting tested. Our approach accommodates for temporal changes in the number of tests being performed and age-specific probabilities to be detected (associated with the probability to develop a clinical form of COVID-19) and assumes a constant prevalence of symptoms suggestive of COVID-19 that cannot be attributed to a SARS-CoV-2 infection. Using this framework to study the epidemics during wintertime where other respiratory viruses might be circulating would require further development.

While shielding older individuals can reduce COVID-19 mortality and morbidity, the intervention would not allow an important relaxation of control measures for other age groups in the absence of vaccines due to the porosity of SARS-CoV-2 transmission across age groups. Pandemic control requires an effort from all age groups.

## Methods

### Hospitalization data

We use hospitalization data extracted from the SI-VIC database. This database is maintained by the ANS (Agence du Numérique en Santé) and provides real-time information on the COVID-19 patients hospitalized in public and private French hospitals. Data, including age, hospitalization date, outcome, and region, are sent daily to Santé Publique France, the French national public health agency. All COVID-19 cases are either biologically confirmed or present with a computed tomographic image highly suggestive of SARS-CoV-2 infection. Missing ages are imputed assuming that the age distribution of newly hospitalized patients for a given week in a given region is similar to the age distribution obtained from patients with age information. Over our study period, the proportion of individuals with missing ages accounted for less than 0.5% of hospitalizations. We restrict our analysis to patients that are hospitalized in general ward beds (Hospitalisation conventionnelle) or ICU beds (Hospitalisation réanimatoire: réanimation, soins intensifs et unité de surveillance continue) and discard patients that are hospitalized in emergency care units (Soins d’urgence), psychiatric care (Hospitalisation psychiatrique), or long-term and rehabilitation care (Soins de suite et réadaptation). We consider events (hospitalizations, transfers, deaths, or discharges) by date of occurrence and correct observed data for reporting delays^1^.

### Test data

SIDEP (Système d’Information de Dépistage Populationnel—Information system for population-based testing) is a national surveillance system describing RT-PCR and antigen test results for SARS-CoV-2 arising from all private and public French laboratories. For the time window used in this analysis (see Supplementary materials), antigen tests were not included in the database. Anonymized data are transmitted daily to Santé Publique France, the French national public health agency, through a secured platform. Upon testing, individuals are asked to report whether they are experiencing symptoms. The test results are reported by date of nasopharyngeal swab and include patient information such as age, delay since symptoms onset, and postal code of the home address. When the home address is not available, the postal code of the lab performing testing is indicated. In case of multiple swabs for a single patient, if the test results are both positive and negative, the first test with positive results is kept. If all the test results are negative, the results of the first test are kept. The number of tests reported in the SIDEP surveillance system for metropolitan France increased throughout summer from 208,214 on the week of 15 June 2020 to 1,115,644 on the week of 14 September 2020 (Supplementary Fig. 29).

### Social contact data

We extracted social contact information from SocialCov, an online survey where participants aged ≥18 yr are invited to describe the contacts they had during the previous day. In the survey, a contact was defined as either a physical contact (e.g., a kiss or a handshake) or a close contact (e.g., face to face conversation at less than 1 meter). Collected information includes the age of the person involved in the contact and the setting where the contact happened (i.e., work, home, leisure place, or others). In addition, respondents living with one or more minors were asked to provide the same information for one of them. The survey was advertised following the same approach as in^17^. Data were collected in accordance with the regulation in force in France for the protection and security of personal data. The answers of 1295 participants were collected between 30 July and 27 September 2020. To comply with the constraints in the survey design of the COMES-F study^41^, used here as the reference for the mixing patterns in France, individuals with more than 40 contacts were excluded from this analysis, reducing the population from an initial number of 1628 to 1550 (including the underaged population). For each age group 0–9 y.o., 10–19 y.o., 20–29 y.o., 30–39 y.o., 40–49 y.o., 50–59 y.o., 60–69 y.o., 70–79 y.o., and ≥80 y.o., we computed the mean daily number of contacts, see Supplementary Table 2.

### Transmission model

To describe the dynamics of SARS-CoV-2 in the French population and the trajectories of hospitalized patients, we use an age-stratified deterministic compartmental model whose structure follows the one described in Salje et al.^1^. In short, infectiousness begins on average 4 days after infection. On average 5 days after infection, infected individuals move to the *I* compartment. Symptoms onset occurs upon entry into the *I* compartment for some of the infected individuals. A subset of infected individuals will develop a severe form of the disease and eventually be hospitalized, on average 7 days after developing symptoms. The probability of hospitalization upon infection is age dependent, as estimated in Salje et al.^1^. The model is stratified in *n*_age_ = 9 age groups: 0–9 y.o., 10–19 y.o., 20–29 y.o., 30–39 y.o., 40–49 y.o., 50–59 y.o., 60–69 y.o., 70–79 y.o., and ≥80 y.o. The model describes the spread of SARS-CoV-2 in the general population and does not account for the specific transmission patterns observed in elderly homes. We thus remove the population of elderly homes from the population of metropolitan France. The model was coded using the *odin* R package^42^.

### Changes in transmission intensity and contact patterns

Assumptions about contact patterns before 11 May 2020 (i.e., the end of the countrywide lockdown) are similar to the ones used in Salje et al.^1^. The contact matrix describing mixing patterns before the implementation of a countrywide lockdown on 17 March 2020 are extracted from the COMES-F survey^41^. During the lockdown, the contact matrix was modified to account for the strict measures put in place. We assume a new change in the reproduction number and in contact patterns on 11 May 2020, when restrictive measures started to be progressively lifted. We also assume another change in transmission on a date that depends on the region (Supplementary Table 3), in line with the observed increase in the proportion of positive tests at the regional level (Fig. 1). For these two post-lockdown time periods, we estimate reproduction numbers (*R*_postLock_ and *R*_rebound_) for each region. At the national level, this corresponds to a reproduction number of 2.90 before 17 March 2020 that was subsequently reduced to 0.67 during the lockdown^1^.

### Modeling contact patterns between the different age groups

Let ${{c}_{i,j}}^{{{{{{{\mathrm{baseline}}}}}}}}$ denote the mean daily number of contacts that an individual aged *i* had with an individual aged *j* in the pre-lockdown period. These values are extracted from the COMES-F survey^41^. Let α_*i*_ denote the reduction of contacts for individuals aged *i* during a time period of interest. To ensure that the total number of contacts between individuals aged *i* and individuals aged *j* is equal to the total number of contacts between individuals aged *j* and individuals aged *i* in the population, we assume that the reduction of contacts between age groups *i* and *j* is equal to *r*_*i,j*_ = min (α_*i*_, α_*j*_). The mean daily number of contacts that an individual aged *i* has with individuals aged *j* is thus equal to ${r}_{i,j}\cdot {{c}_{i,j}}^{{{{{{{\mathrm{baseline}}}}}}}}$. As we are working with normalized contact matrices (i.e., contact matrices divided by their maximum eigenvalue), we are only interested in the relative reduction between different age groups. We thus set α_20-29yr_ = 1 and do not constrain the other α_*i*_ values to be lower than 1.

We assume that contact patterns changed at two distinct periods: first, with the progressive easing of control measures after 11 May 2020 and second at the time of the epidemic rebound (Supplementary Table 3). We estimate parameters related to the reduction of contacts for age groups: 0–9 y.o.;10–19 y.o.; 30–39 y.o.; 40–49 y.o.; 50–59 y.o.; 60–69 y.o.;70–79 y.o.; and ≥80 y.o. for each of the two time periods. We assume that parameters describing the change in mixing patterns from the easing of the lockdown until the rebound are the same in all regions and that mixing patterns during the rebound are region specific.

### Estimating effective contact rates between age groups from the modified matrices

Let ${C}^{{{{{{{\mathrm{rebound}}}}}}}}=({{c}_{i,j}}^{{{{{{{\mathrm{rebound}}}}}}}})$denote the contact matrix estimated for the rebound period. Numerous factors, including changing climate conditions, more outdoor activities or the adoption of protective behaviors such as masks or hand hygiene, can have an impact on the transmission risk associated with a contact with an infected individual (i.e., the transmission rate). We fix the value of the mean daily number of contacts of individuals aged 20–29 yr to the one reported in the SocialCov survey during summer. Let μ^SocialCov^ denote the mean daily number of contacts of individuals aged 20–29 yr reported in the SocialCov survey^17^. We then estimate the mean daily number of contacts that an individual aged *i* has with individuals aged *j* during the rebound period ${{c}_{i,j}}^{{{{{{{\mathrm{eff}}}}}}}}$by

1 $${{c}_{i,j}}^{{{{{{\mathrm{eff}}}}}}}=\frac{{\mu }^{{{{{{\mathrm{SocialCov}}}}}}}}{{\sum }_{j}{{c}_{20-29,j}}^{{{{{{\mathrm{rebound}}}}}}}}\cdot {{c}_{i,j}}^{{{{{{\mathrm{rebound}}}}}}}$$

This rescaling enables a direct interpretation of the coefficients ${{c}_{i,j}}^{{{{{{{\mathrm{eff}}}}}}}}$ as a number of daily contacts. The number of effective contacts in age group *i* can then be derived as

2 $${C}^{{{{{{\mathrm{eff}}}}}}}=\frac{{{\mu }_{20,29}}^{{{{{{\mathrm{SocialCov}}}}}}}}{{\sum }_{j}{{c}_{20-29,j}}^{{{{{{\mathrm{rebound}}}}}}}}\cdot \mathop{\sum}\limits_{j}{{c}_{i,j}}^{{{{{{\mathrm{rebound}}}}}}}$$

which can be interpreted as the model predicted average number of daily contacts between individuals according to age classes. Importantly, the relative contributions of individuals in different age classes are independent of the chosen rescaling.

### Statistical framework

Models are calibrated on weekly age-stratified hospital admissions and number of positive tests among symptomatic individuals in a Bayesian Markov Chain Monte Carlo framework. We account for age-specific probabilities to develop symptoms upon SARS-CoV-2 infection and thus the fact that a greater proportion of all infections are detected among symptomatic individuals. From this, we infer region-specific changes in transmission intensity and contact patterns.

To reduce the impact of potential changes in testing policies, we calibrate our model on the proportion of positive tests amongst symptomatic individuals being tested. Let *S*_+_(*t*, *a*) and *S*_−*t*, *a*_ denote, respectively, the number of positive and negative symptomatic individuals in the population of age *a* at time *t*. We assume that *S*_−*t*, *a*_ is constant over time. Let *p*(*a*) denote the probability of being symptomatic upon SARS-CoV-2 infection amongst individuals aged *a*. Let *N*(*a*) denote the number of individuals aged *a*. Let *I*(*t*, *a*) denote the number of individuals aged *a* in compartment *I* predicted by the model.

The proportion of positive tests among symptomatic individuals of age *a* that were tested is

3 $${P}_{+}(t,\,a)=\frac{{S}_{+}(t,a)}{{S}_{+}(t,a)+{S}_{-}(t,a)}=\frac{p(a)\cdot I(t,a)}{p(a)\cdot I(t,a)+{S}_{-}(t,a)}=\frac{p(a)\cdot I(t,a)}{p(a)\cdot I(t,a)+{\pi }_{a}\cdot N(a)}$$

where π_*a*_ (*a* parameter to be estimated) is the prevalence of symptoms suggestive of COVID-19 that cannot be attributed to a SARS-CoV-2 infection in individuals aged *a* at time *t*. We assume that π_*a*_ is constant across age groups and regions as well as over time. We use the notation π to refer to this quantity. The assumption that π is constant over time is broadly motivated by the low levels of circulation for other respiratory viruses during summer^43–45^. Furthermore, we assume a 3 days delay between symptoms onset and testing, in line with the reported delay between symptoms onset and date of test (Supplementary Fig. 30). We use probabilities to develop a symptomatic form of COVID-19 upon infection as a function of age estimated in Davies et al.^21^.

Further information about the inference procedure is detailed in the Supplement.

### Simulation of intervention strategies targeting single age-groups

We run forward simulations to evaluate the impact of social distancing strategies that reduce contacts in targeted age-groups, starting from the region-specific date of end of calibration. We assume that when an individual reduces his/her contacts, such a reduction is homogeneously distributed across contacts with the different age groups. For a strategy targeting age-groups, a corresponding to a reduction of *x* contacts, we define a new contact matrix as

4 $${C}^{{{{{{{\mathrm{interv}}}}}}}}=({{c}_{i,j}}^{{{{{{{\mathrm{interv}}}}}}}})=({\min }({{\alpha }_{i}}^{{{{{{{\mathrm{interv}}}}}}}},{{\alpha }_{j}}^{{{{{{{\mathrm{interv}}}}}}}})\cdot {{c}_{i,j}}^{{{{{{{\mathrm{eff}}}}}}}})$$

With ${{\alpha }_{i}}^{{{{{{\rm{interv}}}}}}}=\frac{({\sum }_{j}{{c}_{a,j}}^{{{{{{\mathrm{eff}}}}}}})-x}{({\sum }_{j}{{c}_{a,j}}^{{{{{{\mathrm{eff}}}}}}})}$ if *i* = *a* and ${{\alpha }_{i}}^{{{{{{{\mathrm{interv}}}}}}}}=1$ otherwise.

We explore the impact of such intervention strategies on the peak in new infections, the peak in hospital and ICU admissions, the number of deaths arising after the date of change in contact patterns, as well as the life-years lost and QALYs lost after the date where the intervention reducing the number of contacts is implemented. We run a range of scenarios characterized by the effective reproduction number at the time targeted measures are implemented, which corresponds to the region-specific date of end of calibration (Supplementary Table 4). Scenarios are simulated until 1 January 2022. For each one of them, we compute the peak in daily new infections, hospitalizations and admissions in ICUs as well as the number of deaths arising from infections occurring after the date of change in contact patterns and the corresponding number of years of life lost and quality-adjusted life-years lost until the end of the simulation (see Supplementary materials). We explore the impact of interventions in all metropolitan French regions except Corsica due to the high uncertainty around estimates.

### Parametrization of shielding scenarios

For strategies shielding the elderly population, we evaluate the impact of a reduction of 30% and 50% of contacts in those aged 70 yr and above. We also conduct a sensitivity analysis where contacts are reduced in those aged 60 yr and older (Supplementary Fig. 7). We considered the shielding of those aged 70 yr and above to be a more realistic scenario as (i) a non-negligible fraction of those aged 60–69 yr is not retired and remains in the active population^46^, so that reducing contacts in this age group by 50% might be complicated, and as (ii) their perception of their own risk of being susceptible to develop a severe form of COVID-19 might be lower^47^. The value of 50% for the reductions in contact was deliberately defined as an “extreme” scenario to assess the impact of shielding. In Auvergne-Rhône-Alpes, we indeed estimated that individuals aged 80 yr and older have on average 2.9 (2.4–3.4) effective contacts per day (Fig. 2B). A reduction of 50% would bring this number to 1.5 (1.2–1.7). This is below the number of contacts measured during the stringent lockdown implemented in March–May 2020 in metropolitan France^17^. This is also below the mean daily number of contacts measured in the household setting during the pre-pandemic era (1.84 reported in the COMES-F contact survey from Béraud et al.^41^). Reaching such levels of reductions would already appear difficult given (i) the stringency of the first lockdown implemented in March–May 2020 and (ii) the likely limited reduction in contacts within the household in a scenario of extreme shielding where all other contacts are almost removed. We also explored a less stringent shielding scenario, with a reduction of 30% in effective contacts in the elderly population.

### Parametrization of targeted strategies

For strategies targeted towards different age groups, we evaluate the impact of (i) an absolute reduction in effective contacts (e.g., 1) or (ii) a relative reduction in effective contacts (e.g., 10%). We report the results of absolute reductions in the main text as they are more directly interpretable. We also present the second in a sensitivity analysis as the same relative effort in the different age groups does not correspond to the same reduction in absolute number of contacts. To give some context, the absolute and relative reduction in number of contacts that would be necessary to go from the levels measured in the SocialCov survey during summer 2020 to the levels measured during the first national lockdown^17^ are reported in Supplementary Fig. 13. For example, reductions of 4.8 contacts in the 20–29 y.o. and 2.0 contacts in the 80 y.o. and older would have been necessary to bring the number of contacts in these age groups to levels measured during spring 2020. This would have corresponded to 62% and 56% reductions, respectively. We also present the result of age-targeted strategies as a function of the equivalent number of individuals that would need to be put into lockdown to reach such reductions. The corresponding reductions are derived using the SocialCov survey performed during summer 2020 (Supplementary Table 2) and the one performed during the first lockdown in spring 2020^17^.

### Sensitivity analyses

To assess the robustness of our findings, we explore a range of sensitivity analyses:

- Assuming a different susceptibility to SARS-CoV-2 infection between age groups^21^
- Assuming a different susceptibility to SARS-CoV-2 infection and infectivity between age groups^21^
- Assuming a lower susceptibility of 0–19 y.o. compared to 20 y.o. and older^19^
- Including the population of elderly homes in the study population
- Assuming quadratic reductions in contact patterns (i.e., contact reductions apply both to the contacted and the contacting groups)
- Assuming contact patterns are only modified outside the household

Further details about the parametrization of the different sensitivity analyses are reported in the Supplement.

### Ethical considerations

For hospitalization and test data, only anonymized aggregated data were used. As such, no ethical approval was required. The SocialCov contact survey did not qualify as research on human subjects, because the collected data do not allow to identify directly or indirectly the participants in the survey, and was thus exempted from ethical approval.

### Reporting summary

Further information on research design is available in the Nature Research Reporting Summary linked to this article.

## Supplementary information

Supplementary information.

Reporting summary.

## Data Availability

The regional test and hospitalization data used in the analysis are available on Zenodo (10.5281/zenodo.5589952). The aggregated contact data used in our analysis to document to mean number of contacts in the different age groups are reported in the Supplementary information.

## Code availability

The code used to calibrate the model and run forward simulations is available on Zenodo (10.5281/zenodo.5589952).
