# Supplementary material for: SARS-CoV-2 transmission across age groups in France and implications for control
Source: Nat Commun. 2021 Nov 25;12:6895. doi: 10.1038/s41467-021-27163-1 (PMC8617041; doi:10.1038/s41467-021-27163-1)
Supplement: Supplementary file 1 — Supplementary information. [file 41467_2021_27163_MOESM1_ESM.pdf]

SARS-CoV-2 transmission across age groups in France and implications for control.

Cécile Tran Kiem, Paolo Bosetti, Juliette Paireau, Pascal Crépey, Henrik Salje, Noémie Lefrancq, Arnaud Fontanet, Daniel Benamouzig, Pierre-Yves Boëlle, Jean-Claude Desenclos, Lulla Opatowski, Simon Cauchemez.

Supplementary information

Supplementary methods

Supplementary Note 1

Supplementary Figures 1 to 31

Supplementary Tables 1 to 7

Supplementary methods

Estimating the lag between the increase in the proportion of positive tests in 20-29 y.o. and in those 80 y.o. and older

To compute the lag between the increase in the proportion of positive tests in 20-29 y.o. and in 80 y.o. and older, we defined for each region the origin of time as the first week for which the proportion of symptomatic tests among symptomatic individuals reaches 8%. We then calculate the mean of the proportion of positive amongst symptomatic in 20-29 y.o. and in 80 y.o. and older across all regions. We then compute the lag by minimizing the sum of squared errors between the curves. The sum of squared errors is computed over weeks for which at least 5 regions reached the mean proportion.

Time window used for the model calibration

The SIDEP system was initiated on 13 May 2020 with a progressive increase in the number of laboratories reporting the results (from 4562 on the week of 13 May 2020 to 5447 on the week of 15 June 2020) (Supplementary Figure 31). On the week of 13 May 2020, 17.2% of individuals with a positive test result (without missing information about the presence/absence of symptoms) reported developing symptoms more than 2 weeks prior to the test. From the week of 15 June 2020, this proportion was down to 1.0%. From the week of 15 June 2020, the number of laboratories reporting results in the SIDEP database remains quite stable. From this date, the proportion of tested individuals with a delay between symptoms onset and test greater than 2 weeks also remained constant (Supplementary Figure 29). We thus begin the calibration of our model on test data on the week of 15 June 2020. We fitted our model to the proportion of positive tests among symptomatic individuals as this quantity is most likely less sensitive to contact tracing efficiency in a period where the circulation of other respiratory viruses remains low [1].

Following the increase in the number of positive tests and hospital admissions, control measures have progressively been implemented in some regions, resulting in a decrease in the reproduction number (e.g. Provence-Alpes Côte d’Azur region). As we aim to describe transmission patterns during summer before the implementation of additional measures, we define region-specific final date of calibration (the latest possible date being 27 September 2020) based on the time-trends of the proportion of positive tests among symptomatic individuals (Supplementary Table 4).

The age distribution of hospital admissions predicted by our model depends on our assumptions about mixing patterns. Due to the delay between infection and hospital admissions, individuals admitted to hospital during the two weeks following lockdown release will have mostly been infected during the lockdown period. As we fix the contact matrix describing age-specific contact patterns during the lockdown, we only begin the calibration of our model on age-stratified data on 25 May 2020 (i.e. 2 weeks after the end of the country-wide lockdown). Between 11 May 2020 and 24 May 2020, we calibrate our model on the daily number of hospital admissions occurring in each metropolitan French region.

Models are calibrated using SI-VIC data (extracted from the SI-VIC database on 12 October 2020) between 11 May 2020 and the region-specific final date of calibration and on the weekly proportion of positive tests among individuals reporting symptoms (extracted from SIDEP data) between 15 June 2020 and the region-specific final date of calibration.

Computing the effective reproduction number in an age-structured population

The basic reproduction number R_0 corresponds to the average number of infections resulting from a typical index case in a completely susceptible population in the absence of intervention. We introduce the intervention reproduction number R_i to describe the impact of interventions, behavioural changes or climatic conditions on the value of the transmission rate. This value corresponds to the average number of infections resulting from a typical index case that would be observed in a completely susceptible population under a given set of interventions. The effective reproduction number R_{eff} accounts for the fact that a fraction of the population is immune and no longer contributes to the infection spread. To compute the effective reproduction number, we use the next-generation matrix approach [2]. Let ${p_S}^i(t)$ denote the proportion of the population aged i susceptible to infection at time t. Let $c_{i,j}$ denote the mean daily number of contacts that and individual aged i has with someone aged j.

The effective reproduction number is then derived as:

$$R_{eff}(t) = R_i \cdot \rho(\ [c_{i,j} \cdot {p_S}^j(t)]_{ij}\ ) \ /\ \rho(\ [c_{i,j}]_{ij}\ ) \qquad (1)$$

where $\rho(M)$denotes the spectral radius of a matrix M.

Statistical framework

Models are calibrated using SI-VIC data (extracted from the SI-VIC database on 12 October 2020) between 11 May 2020 and the region-specific final date of calibration and on the weekly proportion of positive tests among individuals reporting symptoms (extracted from SIDEP data) between 15 June 2020 and the region-specific final date of calibration (see Supplementary materials). Parameters are estimated using a Bayesian Markov Chain Monte Carlo framework. We develop a Metropolis-Hastings algorithm with lognormal proposals and uniform priors for all the parameters. Chains are run with 100,000 iterations removing 5,000 iterations of burn-in.

Let ${Adm_{hosp}}^{obs}(t)$ and ${Adm_{hosp}}^{pred}(t)$ denote the observed and expected number of COVID-19 hospital admissions on day t for the whole population. After 24 May 2020, age-groups are specifically considered and data are aggregated at the week level. Let ${Adm_{hosp}}^{obs}(w,a)$ and ${Adm_{hosp}}^{pred}(w,a)$ denote the observed and predicted number of COVID-19 patients belonging to age group a admitted to hospital on week w. Let $X^{obs}(w,a)$ and $N^{obs}(w,a)$ denote the number of positive tests and the number of tests amongst symptomatic individuals being tested on week w in age-group a. Let ${P_+}^{pred}(w,a)$ denote the proportion of positive tests amongst symptomatic

individuals tested predicted by the model for age group a on week w. The likelihood function until day T is then defined as:

$$L_T = L^{hosp}(T) \cdot L^{Age-Hosp}(T) \cdot L^{Age-Tests}(T) \quad (2)$$

with:

$$L^{hosp}(T) = \prod_{t=11\,May}^{24\,May} g_{\delta_1}(Adm_{hosp}{}^{obs}(t) | Adm_{hosp}{}^{pred}(t)) \quad (3)$$

$$L^{Age-Hosp}(T) = \prod_{w=w_1}^{w_T} \prod_{a=1}^{n_{age}} g_{\delta_2}(Adm_{hosp}{}^{obs}(w,a) | Adm_{hosp}{}^{pred}(w,a)) \quad (4)$$

$$L^{Age-Tests}(T) = \prod_{w=w_2}^{w_T} \prod_{a=1}^{n_{age}} g_{\delta_3}(X^{obs}(w,a) | N^{obs}(w,a) \cdot P_+{}^{pred}(w,a)) \quad (5)$$

Where $w_1$corresponds to the week starting on 25 May 2020, w_T corresponds to the week of time T, $w_2$corresponds to the first week for which we consider test data to be reliable (15 June 2020), $g_\delta(\cdot|X)$ is a negative binomial distribution of mean X and overdispersion parameter X^δ. n_{age} corresponds to the number of age groups in the model. δ_2 and δ_3 are overdispersion parameters to be estimated. $\delta_1$is the value of the overdispersion parameter estimated during the first wave of SARS-CoV-2 in France [3].

Computing age-specific probability of ICU admission and death given hospitalization

To capture changes in the probability of ICU admission given hospitalisation and death given hospitalisation of COVID-19 patients in Metropolitan France [4], we compute updated estimates from the proportion of patients in the different age groups that have been admitted in ICU or that died in September-October 2020 reported in the SI-VIC surveillance system (Supplementary Table 5). Using the same approach, we compute the proportion of deaths that occur in ICU in the different age groups (Supplementary Table 6).

Computing the peak in ICU admissions, the number of deaths, years of life lost and quality adjusted years of life lost arising from infections occurring after the date of change in contacts patterns

Based on the age-specific probabilities of death given hospitalization estimated between 13 July 2020 and 30 September 2020 (Supplementary Table 5), we compute the number of deaths arising from infections occurring after the date of change in contact patterns and the corresponding number of years of life lost until the end of the simulation. Life expectancies for a given age group were computed using data from the National Institute for Statistics and Economic Studies (Institut national de la statistique et des études économiques - INSEE) [5]. We also compute the quality adjusted years of life lost arising from infections occurring after the date of change in contact patterns. We use age-specific utilities derived for the French setting [6]. We follow the approach proposed by Sandmann et al. [7] to derive the quality-adjusted life years (QALYs) loss per symptomatic cases, non-fatal hospitalized cases in general wards et non-fatal hospitalized cases

admitted in ICUs. We assume that a symptomatic case results in a loss of 0.008 QALYs [8], a non-fatal hospitalization in general ward beds in a loss of 0.018 QALYs [9,10] and a non-fatal ICU hospitalization in a loss of 0.15 QALYs [11,12]. To compute the number of symptomatic infections, we use the age-specific proportion of clinical infections, as estimated in Davies et al [13]. The corresponding weights used to compute the number of life years lost and quality adjusted life years lost arising from deaths are reported in Supplementary Table 7.

Sensitivity analyses - rationale and description

In the following paragraph, we detail the different sensitivity analyses that we explore alongside a rationale for considering each of them:

→ **Assuming a different susceptibility to SARS-CoV-2 infection between age-groups**

In our baseline scenario, we do not account for a different susceptibility of the different age groups to SARS-CoV-2 infection. In a sensitivity analysis, we explore a scenario with different susceptibilities, using the values estimated by Davies et al. [13]. Let σ_i denote the susceptibility of age group i. For a contact matrix $(c_{i,j})_{i,j}$ describing the average daily number of contacts that individuals of age group i have with individuals of age group j, we modify the coefficients as $(\sigma_i \cdot c_{i,j})_{i,j}$ to account for the susceptibility as a function of age.

→ **Assuming a different susceptibility to SARS-CoV-2 infection and infectivity between age-groups**

In our baseline scenario, we do not account for a different susceptibility of the different age groups to SARS-CoV-2 infection nor for a different infectivity across the different age groups. In a sensitivity analysis, we explore a scenario with different susceptibilities, using the values estimated by Davies et al. [13] and different infectivities for the different age groups. Let σ_i (respectively θ_i) denote the susceptibility (respectively the infectivity) of age group i. For a contact matrix $(c_{i,j})_{i,j}$ describing the average daily number of contacts that individuals of age group i have with individuals of age group j, we modify the coefficients as $(\sigma_i \cdot c_{i,j} \cdot \theta_j)_{i,j}$ to account for susceptibility and infectivity as a function of age. To compute values of the infectivity for different age groups, we assume that symptomatic individuals are more infectious than asymptomatic individuals and that their probability of transmission upon contact with a susceptible individual is $\theta_{asympto} = 55\%$ that of symptomatic individuals [14]. The infectivity of age group j can then be derived as:

$$\theta_j = p_j^{sympto} \cdot (1 - \theta_{asympto}) + \theta_{asympto} \quad (6)$$

where p_j^{sympto} is the probability that an individual in age group $j$develops symptoms upon infection [13].

→ **Assuming a lower susceptibility of 0-19 y.o. compared to 20 y.o. and older**

Children have been suggested to be less susceptible to SARS-CoV-2 infection compared to adults, with younger children being less susceptible than teenagers. Uncertainty remains regarding the extent to which susceptibility increases with age. To further account for this uncertainty, we explore a scenario where children aged 0-9 y.o. are 50% less susceptible as those

20 y.o. and older and children aged 10-19 y.o. are 25% less susceptible than adults aged 20 y.o. and older [15].

For these three scenarios where we vary assumptions about infectivity and susceptibility by age, we derived adjusted contacts from the estimated effective contacts. We define adjusted contacts as the corresponding number of raw contacts assuming the difference in effective and raw contacts can be entirely explained by variations in susceptibility and infectivity in the different age groups. More specifically, let ${c_{i,j}}^{eff}$ denote the mean daily number of effective contacts that an individual aged i has with individuals aged j. Let σ_i (respectively θ_i) denote the susceptibility (respectively the infectivity) of age group i. The adjusted mean daily number of contacts is then derived as:

$${c_{i,j}}^{adj} = \frac{{c_{i,j}}^{eff}}{\sigma_i \cdot \theta_j} \tag{7}$$

→ **Including the population of elderly homes in the study population**

Since the beginning of the pandemic, elderly homes have accounted for a substantial share of the number of COVID-19 deaths in France [16]. As the epidemic dynamics in these locations as well as the structure of contacts is expected to be significantly different than that in the community, we removed the population of elderly homes from the French population for our baseline scenario and we discarded the results of tests from elderly homes residents. The SI-VIC surveillance system does not distinguish from all patients admitted in hospitals following a SARS-CoV-2 infection, those that live in elderly homes. In our baseline scenario, we removed the population of elderly homes from the study population and from the test data used for the calibration. As an indeterminate fraction of hospitalizations reported in the SI-VIC database are likely to be attributable to elderly home residents, we conduct a sensitivity analysis keeping the population of elderly homes in our study population and keeping using the tests results from elderly home patients for our calibration. The choice of this baseline scenario where we removed elderly homes population was motivated by the low share of elderly residents among all individuals admitted to hospital (6.5% from 1 March 2020 to 21 February 2021 ; 11.1% of the 70 y.o. and older assuming all admitted residents are 70 y.o. and older).

→ **Considering quadratic reductions in contact patterns**

In our baseline scenario, we considered linear reduction in contact patterns. For instance, regarding the simulation of strategies targeting different age groups, this meant that when we were considering a reduction of 10% among 20-29 y.o., the contacts of this age group with all other age groups were reduced by 10%. With the same notation as the one used in the methods section, we used the following model:

$$C^{interv} = ({c_{i,j}}^{interv}) = (min({\alpha_i}^{interv}, {\alpha_j}^{interv}) \cdot {c_{i,j}}^{eff}) \tag{8}$$

An alternative to model the impact of different reductions in contact patterns is to consider quadratic reduction in contact patterns. In this case, a reduction of 10% in mobility among 20-29 y.o. would correspond to a 10% reduction in contact between 20-29 y.o. and all other age groups and a reduction of 19% of contacts of 20-29 y.o. with 20-29 y.o. compared to the equation detailed above, we use the following parametrization:

$$C^{interv} = (c_{i,j}{}^{interv}) = (\alpha_i{}^{interv} \cdot \alpha_j{}^{interv} \cdot c_{i,j}{}^{eff}) \quad (9)$$

→ **Assuming contact patterns are only modified outside the household**

In our baseline scenario, we assumed that when an age group reduces their contacts, this affects the contacts of all other age groups homogeneously. Non-pharmaceutical interventions implemented have mostly been targeting contacts outside the household, so that this assumption might not hold for household contacts. Studies have for instance reported that, when interventions were implemented, contacts between school-aged children were removed whereas some contacts with younger adults were maintained (e.g. with parents) [17] . We explore a sensitivity analysis where only contacts outside the household are modified following the same approach as in our baseline scenario (homogeneous reduction outside the household).

Supplementary Note 1

The abbreviations used for the names of the metropolitan French regions are:

ARA: Auvergne-Rhônes-Alpes
BFC: Bourgogne-Franche-Comté
BRE: Bretagne
CVL: Centre Val de Loire
COR: Corse
GES: Grand Est
HDF: Hauts-de-France
IDF: Île-de-France
NAQ: Nouvelle-Aquitaine
NOR: Normandie
OCC: Occitanie
PAC: Provence Alpes Côte d’Azur
PDL: Pays de la Loire

Supplementary materials

Supplementary Figure 1

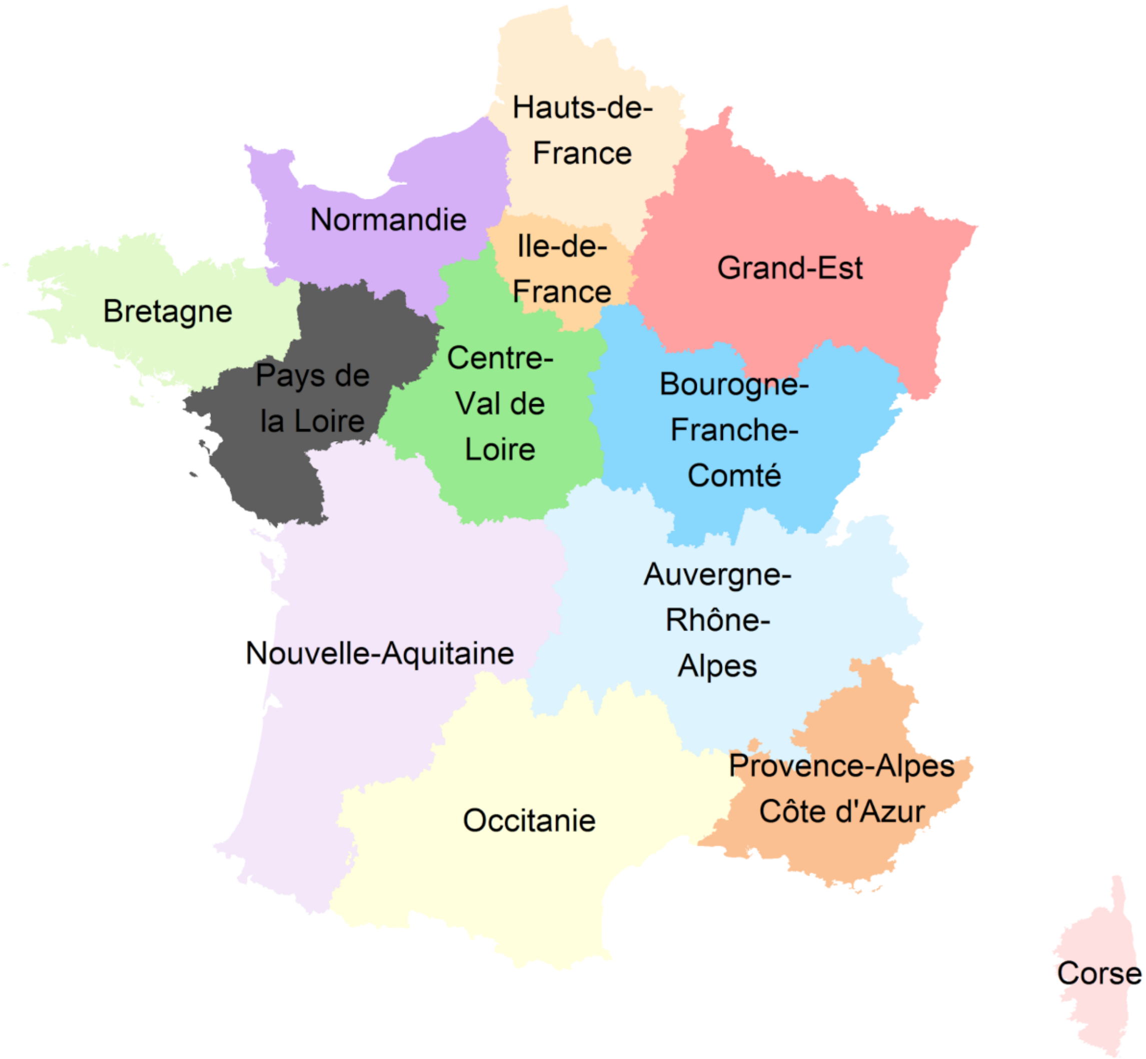

Supplementary Figure 1: Map of the 13 regions of metropolitan France

Supplementary Figure 2

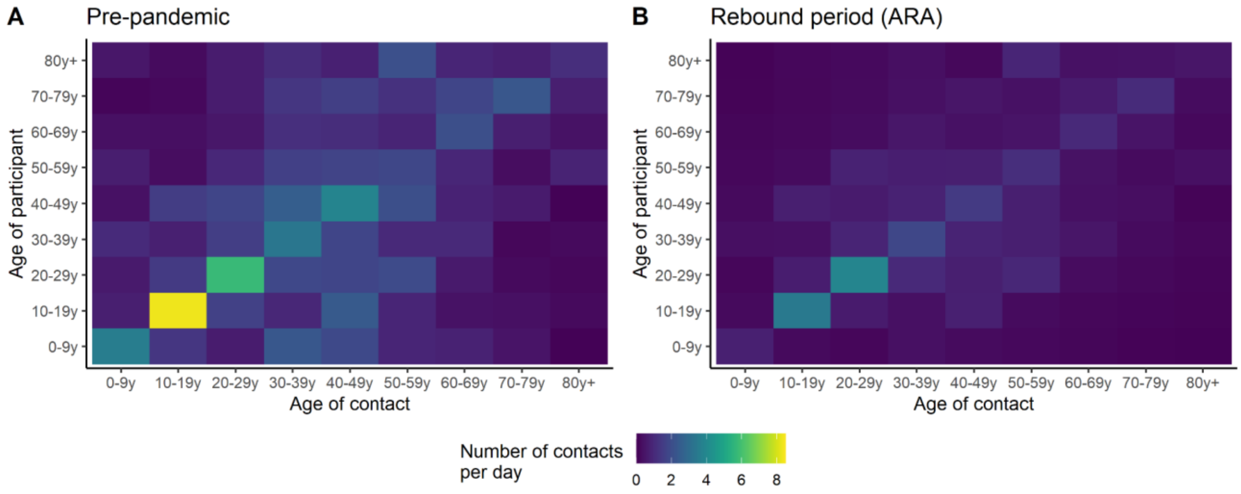

Supplementary Figure 2: Contact matrices across different periods. (A) Contact matrix describing the mixing patterns during the pre-pandemic era [18]. **(B)** Effective contact matrix describing the mixing patterns between July 9th, 2020 and September 28th, 2020 in the Auvergne-Rhône-Alpes region.

Supplementary Figure 3

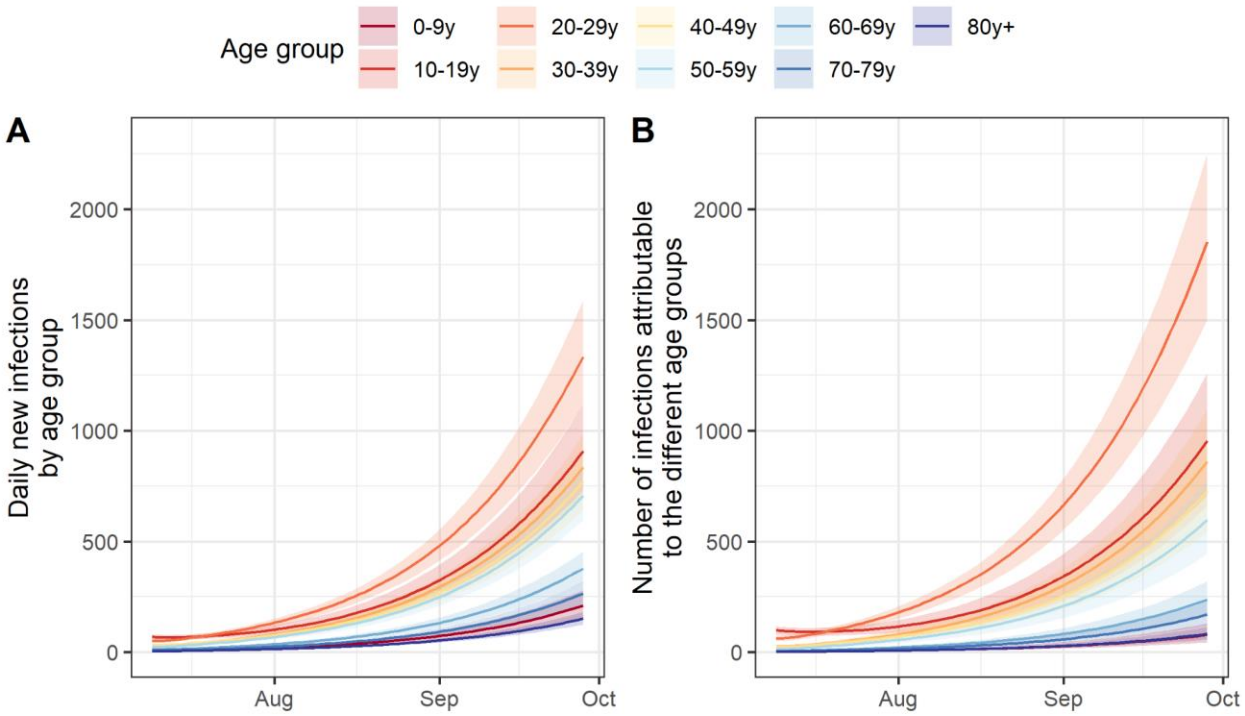

Supplementary Figure 3: Dynamics of infections in the different age groups. (A) Daily new infections by age group. **(B)** Number of daily new infections attributable to the different age groups. The results are reported for the Auvergne-Rhône-Alpes region during the rebound period (9 July-28 September 2020). The lines correspond to the mean values obtained from 500 simulations from the posterior distributions.The shaded areas correspond to 95% credible intervals.

Supplementary Figure 4

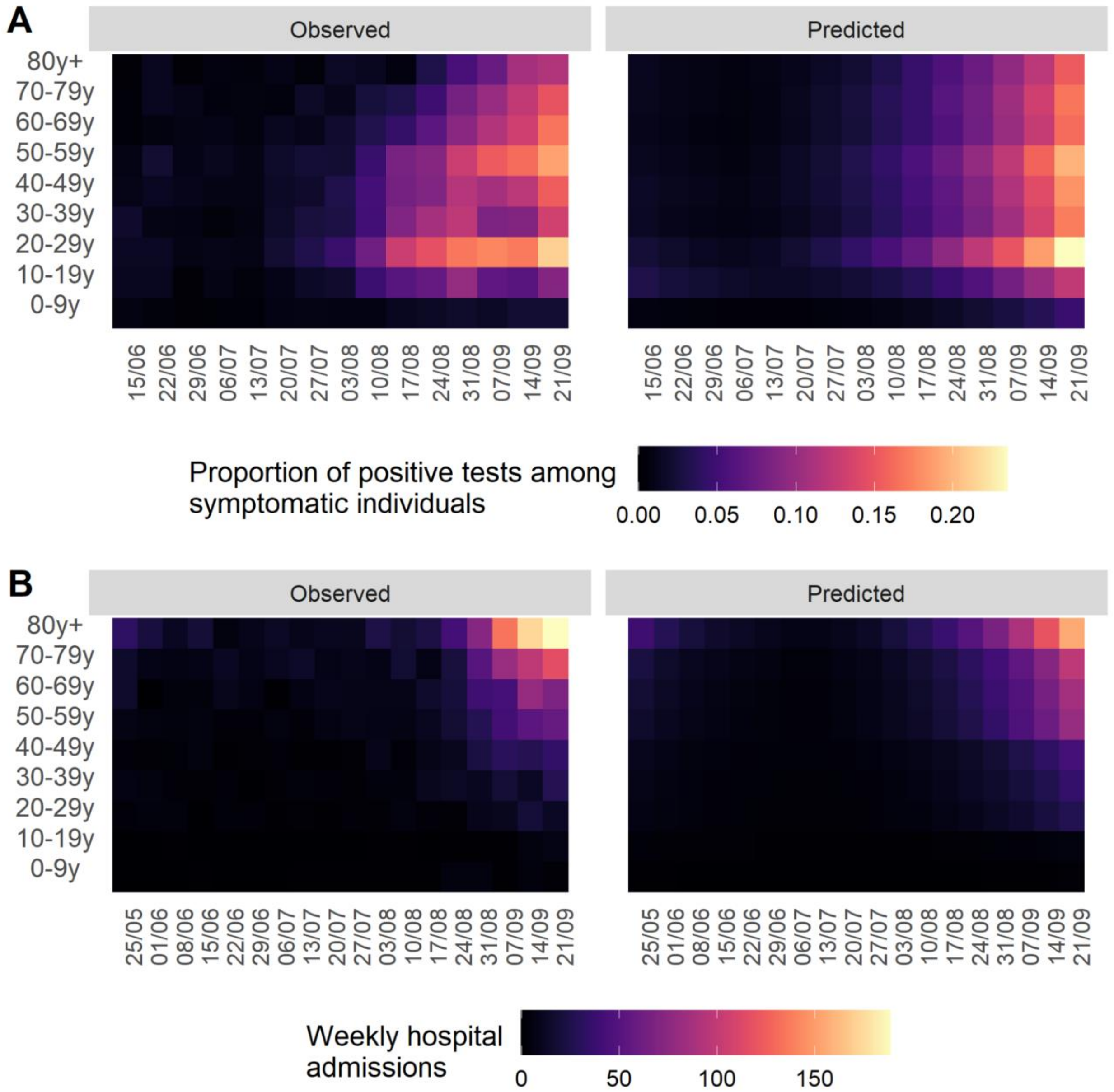

Supplementary Figure 4: Predicted and observed dynamics of the epidemic in Auvergne-Rhône-Alpes across age-groups. (A) Observed and predicted dynamics of the proportion of positive tests among symptomatic individuals tested by age-group. **(B)** Observed and predicted dynamics of the weekly hospital admissions by age-group.

Supplementary Figure 5

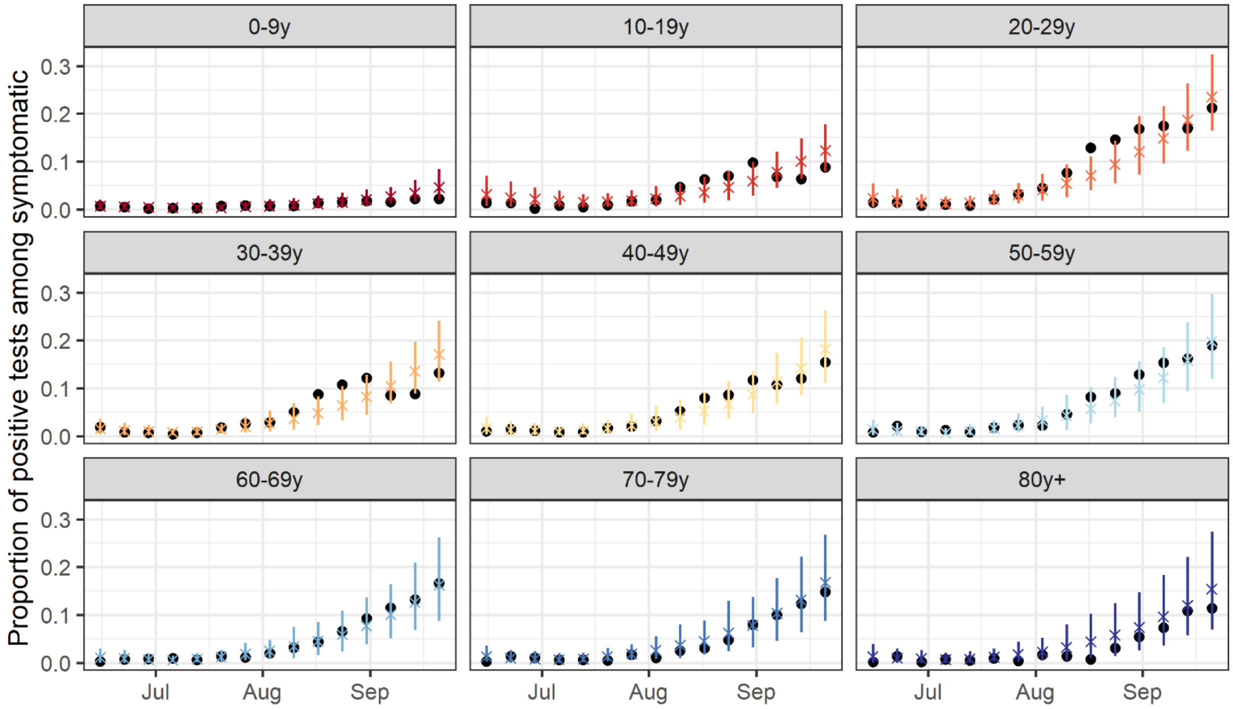

Supplementary Figure 5: Model-predicted and observed proportion of positive tests among symptomatic individuals in Auvergne-Rhône-Alpes by age group. Proportion of positive test among symptomatic individuals aged 0-9 y.o., 10-19 y.o., 20-29 y.o., 30-39 y.o., 40-49 y.o., 50-59 y.o., 60-69 y.o., 70-79 y.o. And over 80 y.o. in Auvergne-Rhône-Alpes. The colored crosses indicate model predictions. The black points indicate the proportions of positive tests among symptomatic individuals extracted from the SIDEP database. The vertical segments indicate 95% credible intervals obtained from 500 simulations from the posterior distribution.

Supplementary Figure 6

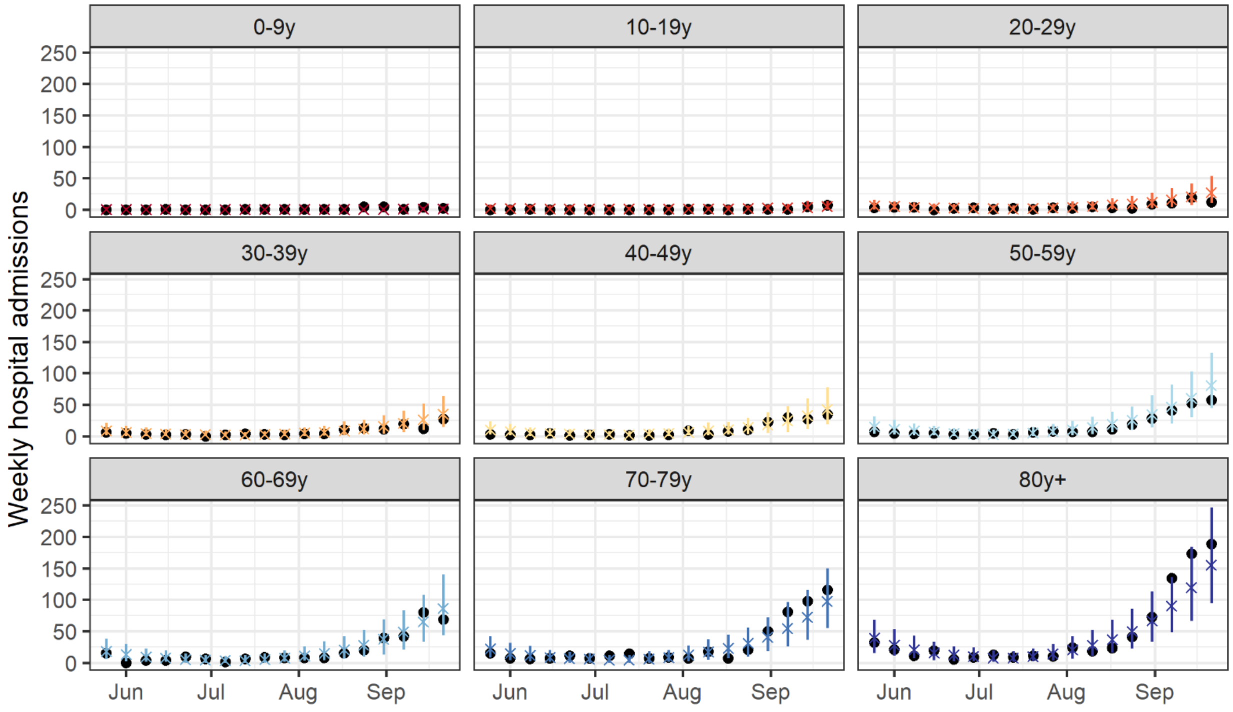

Supplementary Figure 6: Model predicted and observed age-stratified hospital admissions in Auvergne-Rhône-Alpes by age group. Weekly hospital admissions of individuals aged 0-9 y.o., 10-19 y.o., 20-29 y.o., 30-39 y.o., 40-49 y.o., 50-59 y.o., 60-69 y.o., 70-79 y.o. and over 80 y.o. in Auvergne-Rhône-Alpes. The colored crosses and segments indicate model predictions. The black points indicate weekly hospital admissions extracted from the SI-VIC database. The vertical segments indicate 95% credible intervals obtained from 500 simulations from the posterior distribution.

Supplementary Figure 7

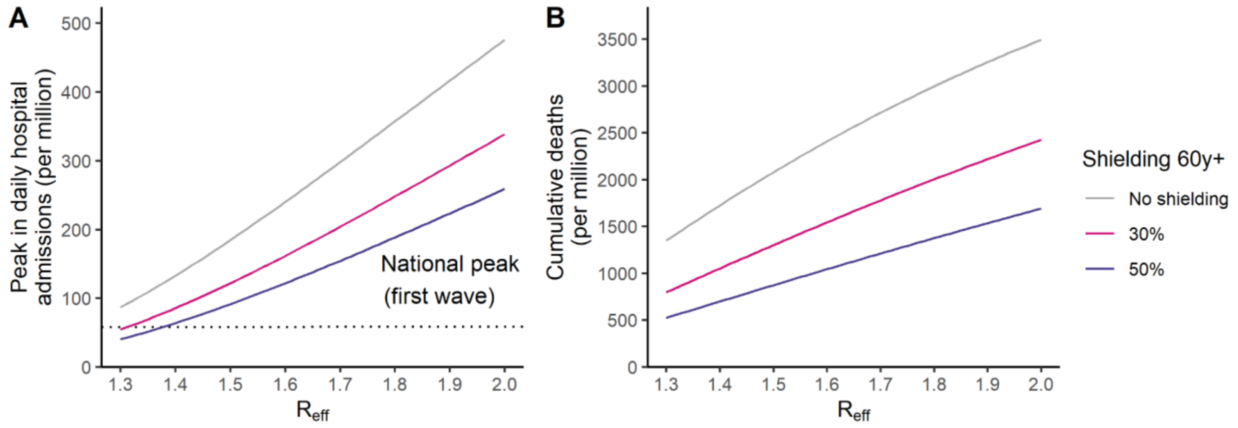

Supplementary Figure 7: Impact of strategies shielding those aged 60 y.o. and above. (A) Peak in hospital admissions per million and **(B)** number of deaths per million as a function of the effective reproduction number R_{eff} assuming a reduction of 50% or 30% in effective contacts of those older than 60 y.o. The number of deaths is computed from the time interventions are implemented until the end of the simulation.

Supplementary Figure 8

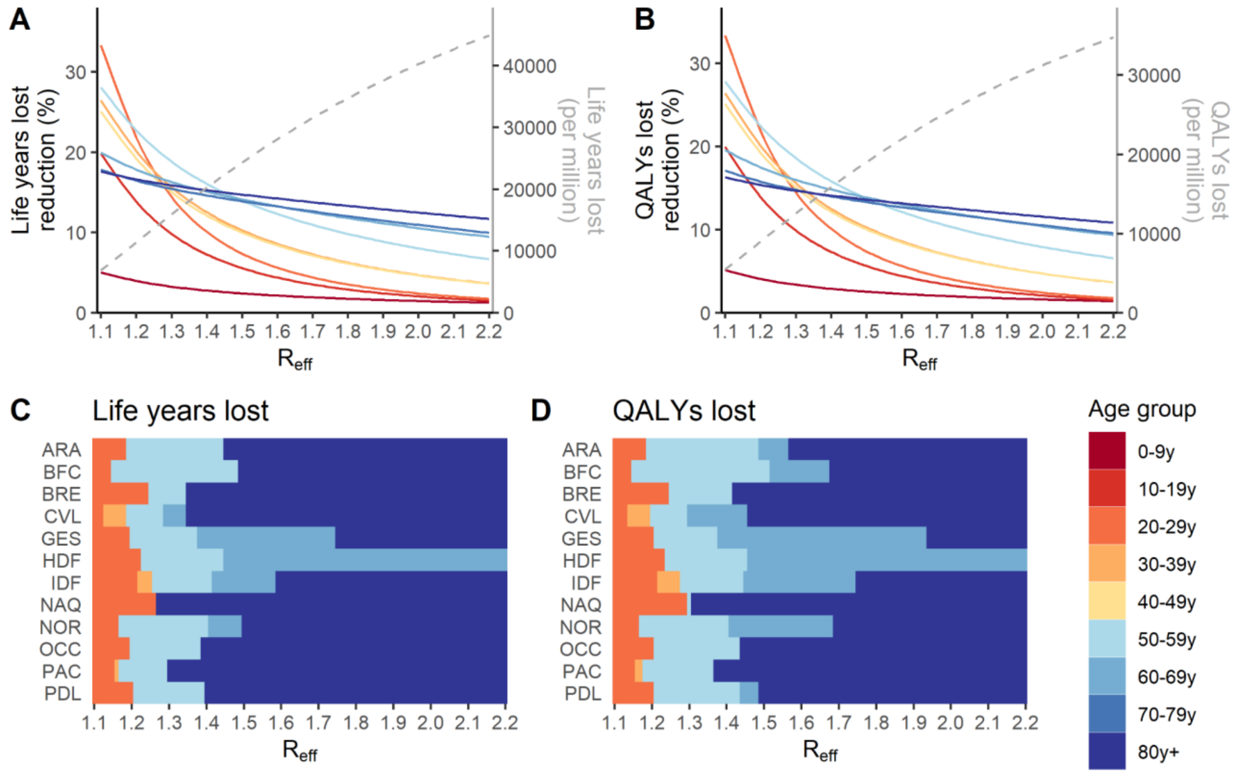

Supplementary Figure 8: Impact of strategies targeting specific age groups on the number of life-years lost. Reduction in **(A)** the number of life-years lost and **(B)** the number of QALYs lost in Auvergne-Rhône-Alpes region as a function of the effective reproduction number R_{eff} when the intervention is implemented for a reduction of 1 contact. The grey dotted lines indicate, in the absence of additional measure, the value of the target metrics. Age-groups for which a reduction of 1 contact results in the highest impact on the reduction of **(C)** the number of life years lost and **(D)** the number of QALYs lost as a function of the effective reproduction number R_{eff}. Region's abbreviations are detailed in Supplementary Note 1.

Supplementary Figure 9

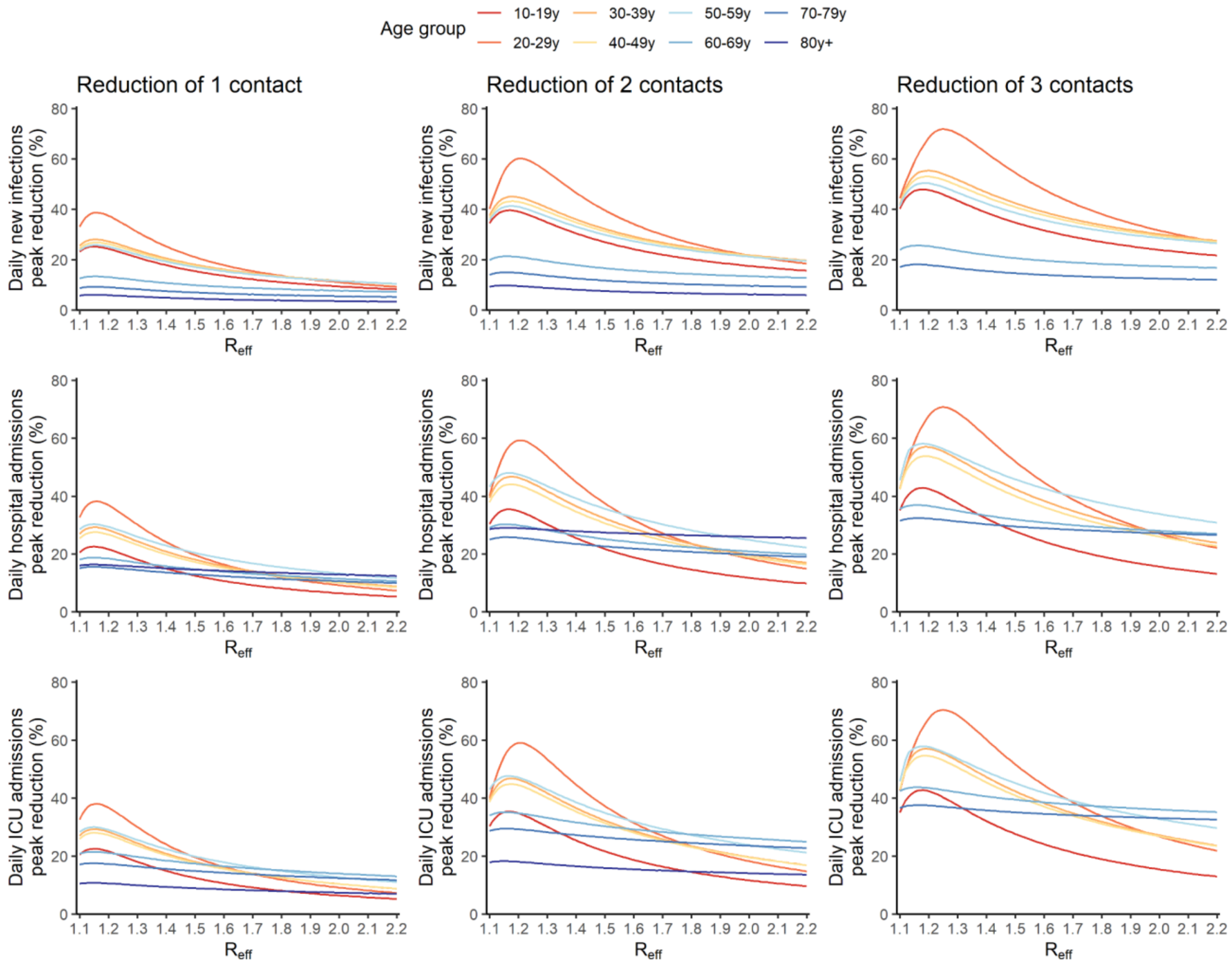

Supplementary Figure 9: Impact of larger reduction of contacts for strategies targeting different age groups in Auvergne-Rhône-Alpes on the peak in daily new infections (first line), the peak in hospital admissions (second line) and the peak in daily ICU admissions (third line) as a function of the effective reproduction number R_{eff} when the intervention is implemented. Results are displayed for a reduction of 1 contact (first column), 2 contacts (second column) and 3 contacts (third column) in the targeted age groups.

Supplementary Figure 10

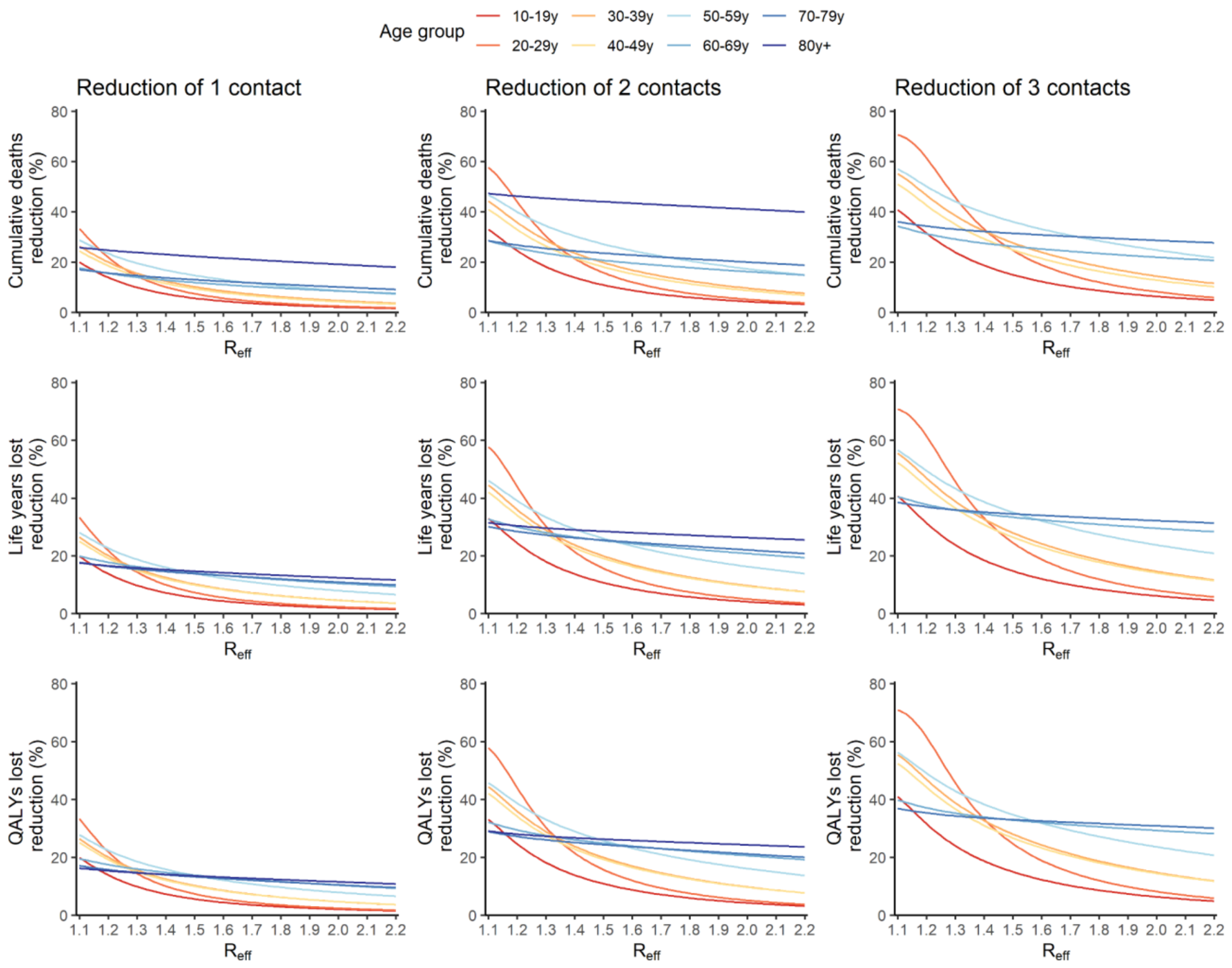

Supplementary Figure 10: Impact of larger reduction of contacts for strategies targeting different age groups in Auvergne-Rhône-Alpes on the number of deaths (first line), life years lost (second line) and QALYs lost (third line) after the implementation of the intervention as a function of the effective reproduction number R_{eff} when the intervention is implemented.

Supplementary Figure 11

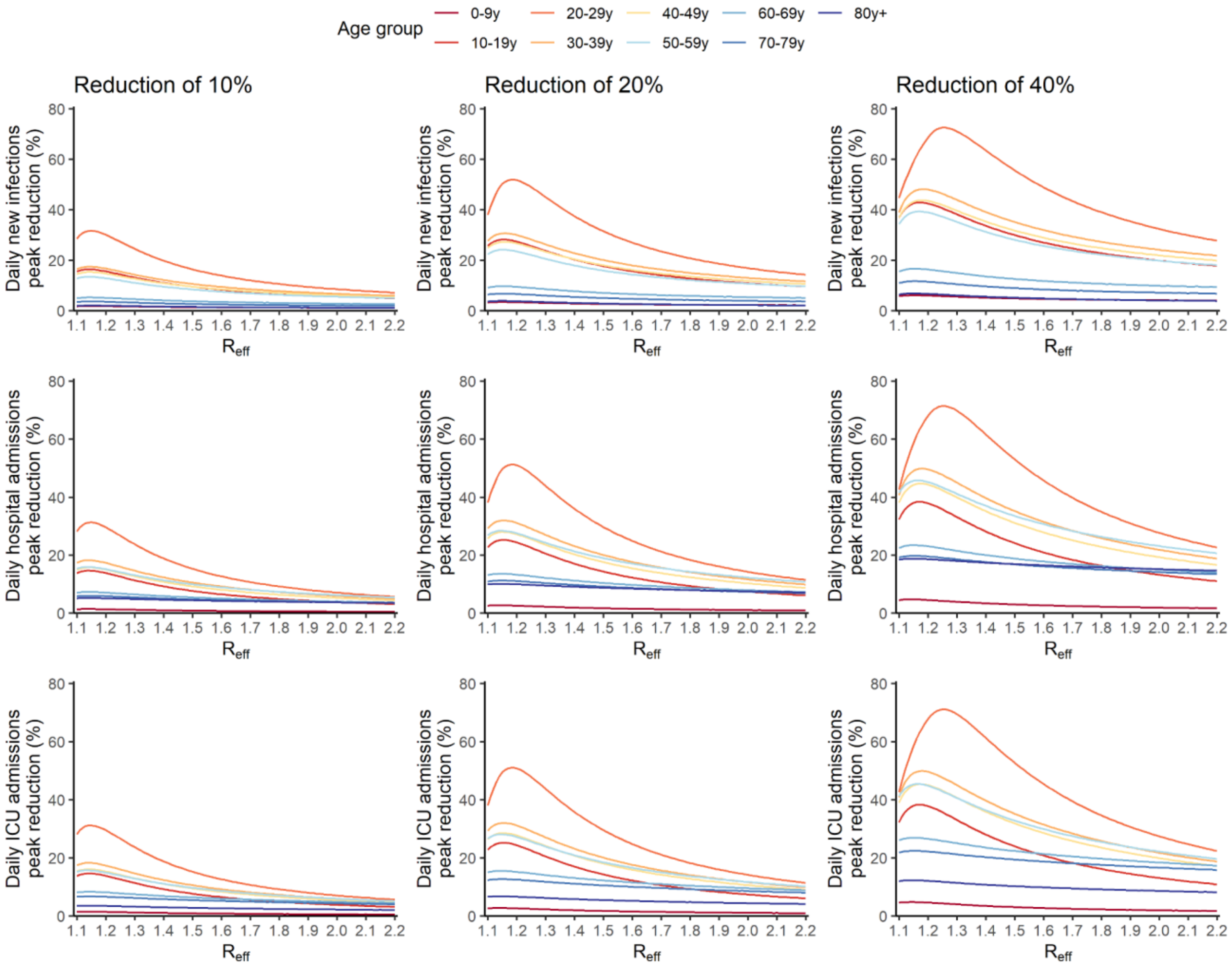

Supplementary Figure 11: Impact of strategies targeting different age groups in Auvergne-Rhône-Alpes on the peak in daily new infections (first line), the peak in hospital admissions (second line) and the peak in daily ICU admissions (third line) as a function of the effective reproduction number R_{eff} when the intervention is implemented. Results are displayed for a reduction of 10% (first column), 20% (second column) and 40% (third column) in the number of contacts of the targeted age groups.

Supplementary Figure 12

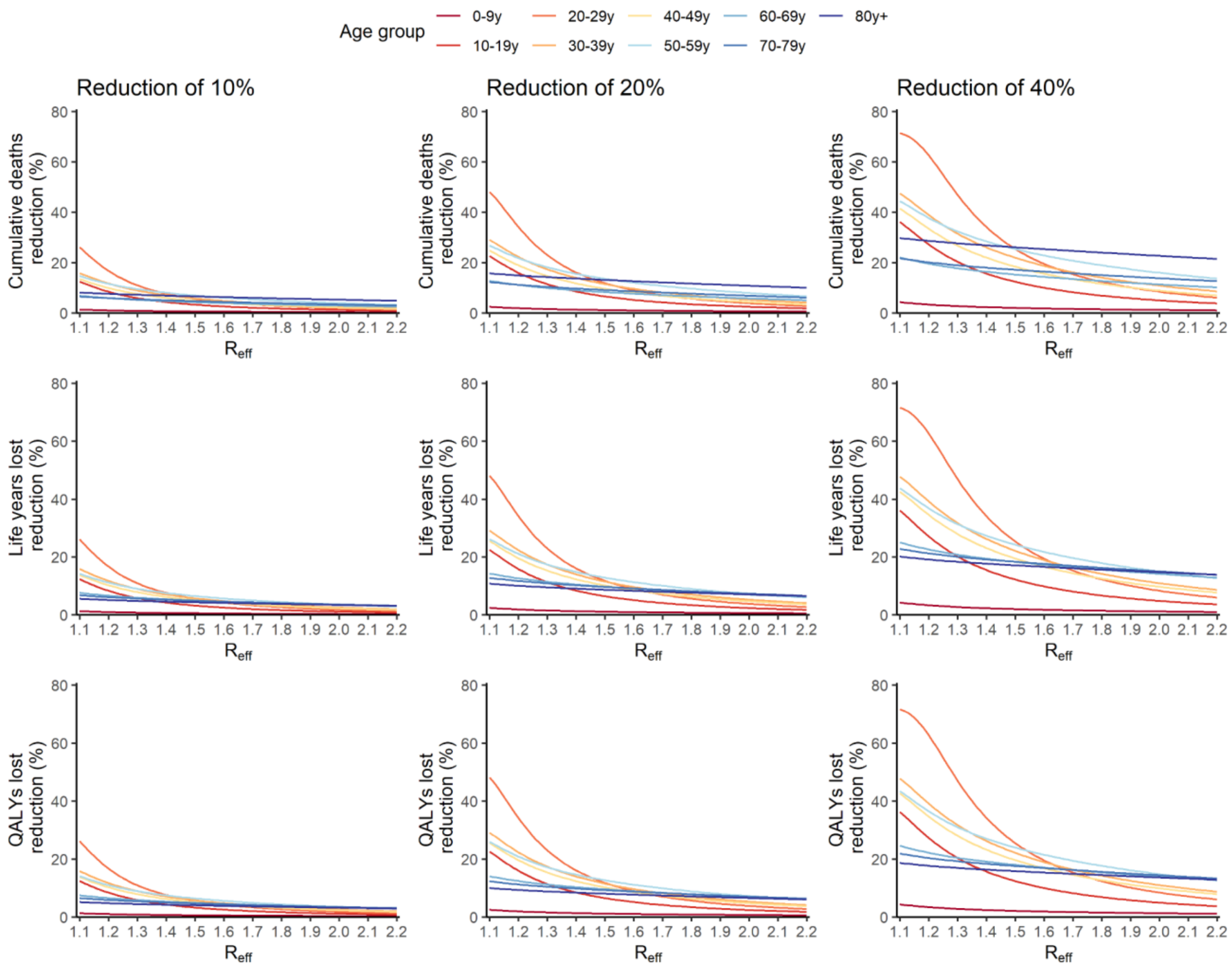

Supplementary Figure 12: Impact of strategies targeting different age groups in Auvergne-Rhône-Alpes on the number of deaths (first line), the life years lost (second line) and the QALYs lost (third line) as a function of the effective reproduction number R_{eff} when the intervention is implemented. Results are displayed for a reduction of 10% (first column), 20% (second column) and 40% (third column) in the number of contacts of the targeted age groups.

Supplementary Figure 13

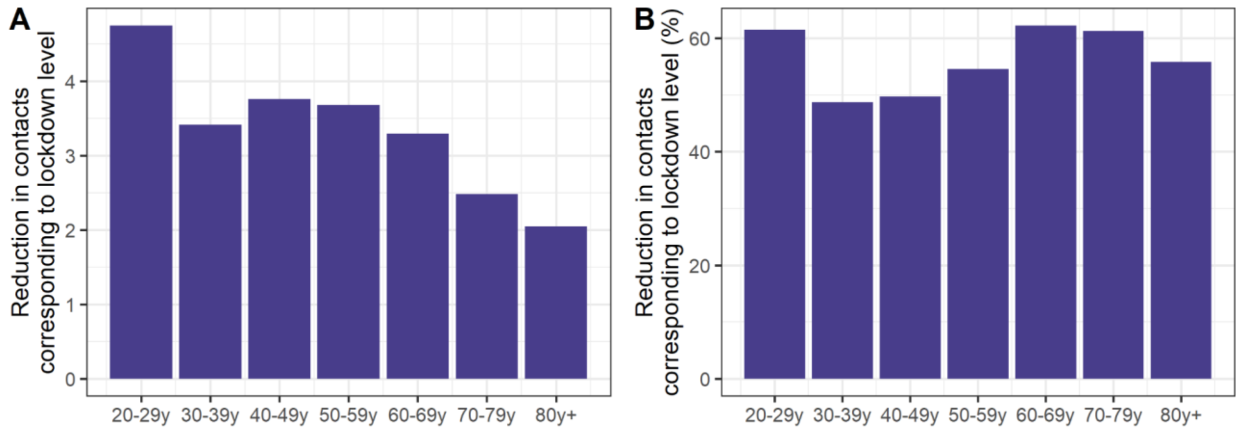

Supplementary Figure 13: Reduction in contacts necessary to move the number of contacts from levels measured during summer 2020 to those measured during the first lockdown of spring 2020. Results are reported both in absolute **(A)** and **(B)** relative reductions. The reductions are computed using the contacts measured in the SocialCov survey during spring 2020 [19] and summer 2020 (Supplementary Table 2).

Legend for Supplementary Figures 14-25

(A) Proportion of positive tests among symptomatic individuals by age group. **(B)** Weekly hospital admissions of individuals by age group. The colored crosses and segments indicate model predictions. The colored crosses and segments indicate model predictions. The black points in panels (A) indicate the proportions of positive tests among symptomatic individuals extracted from the SIDEP database. The black points in panels (B) indicate weekly hospital admissions extracted from the SI-VIC database. The vertical segments indicate 95% credible intervals obtained from 500 simulations from the posterior distribution.

Supplementary Figure 14: Model predictions and observations in Bourgogne-Franche-Comté

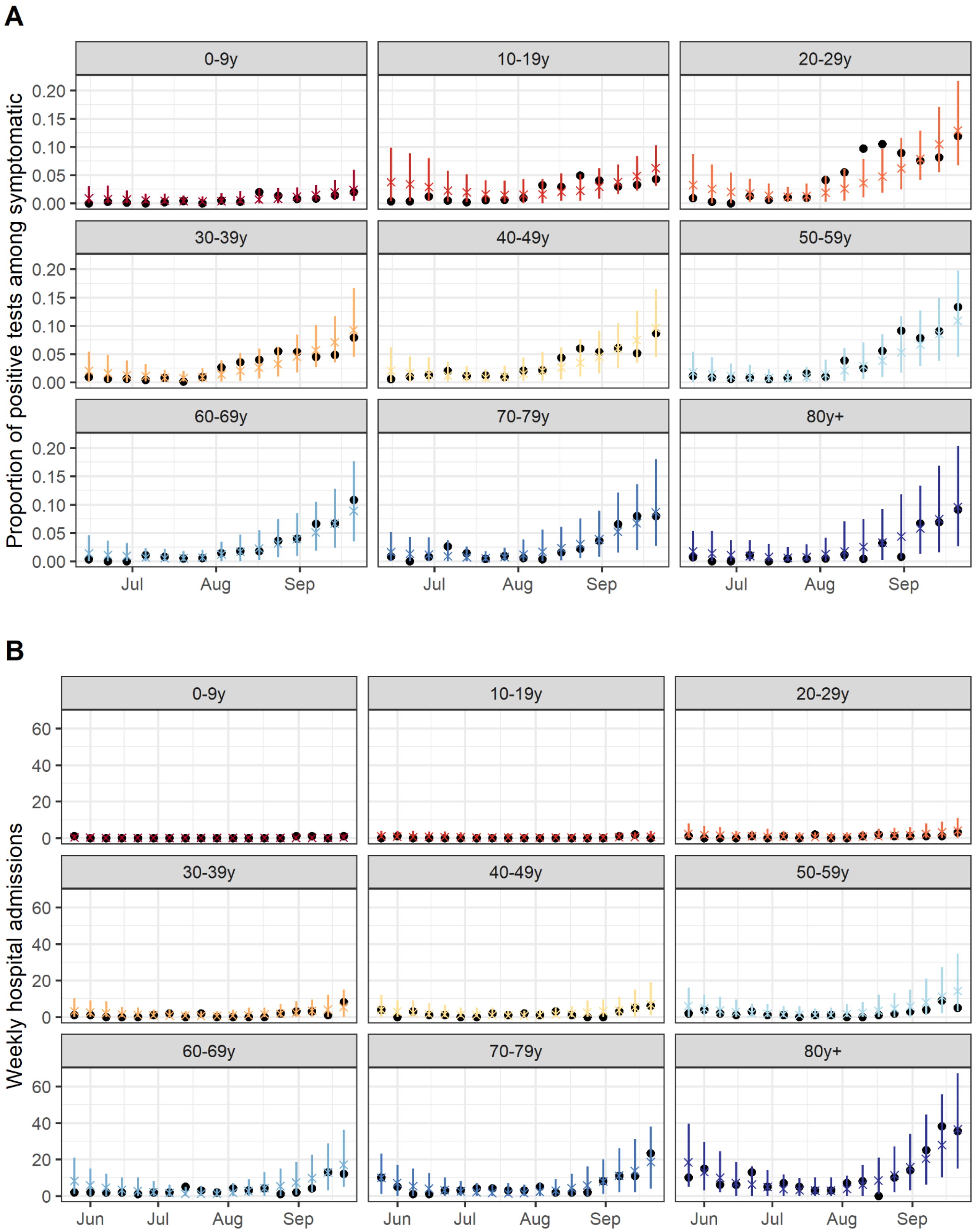

Supplementary Figure 15: Model predictions and observations in the Bretagne region

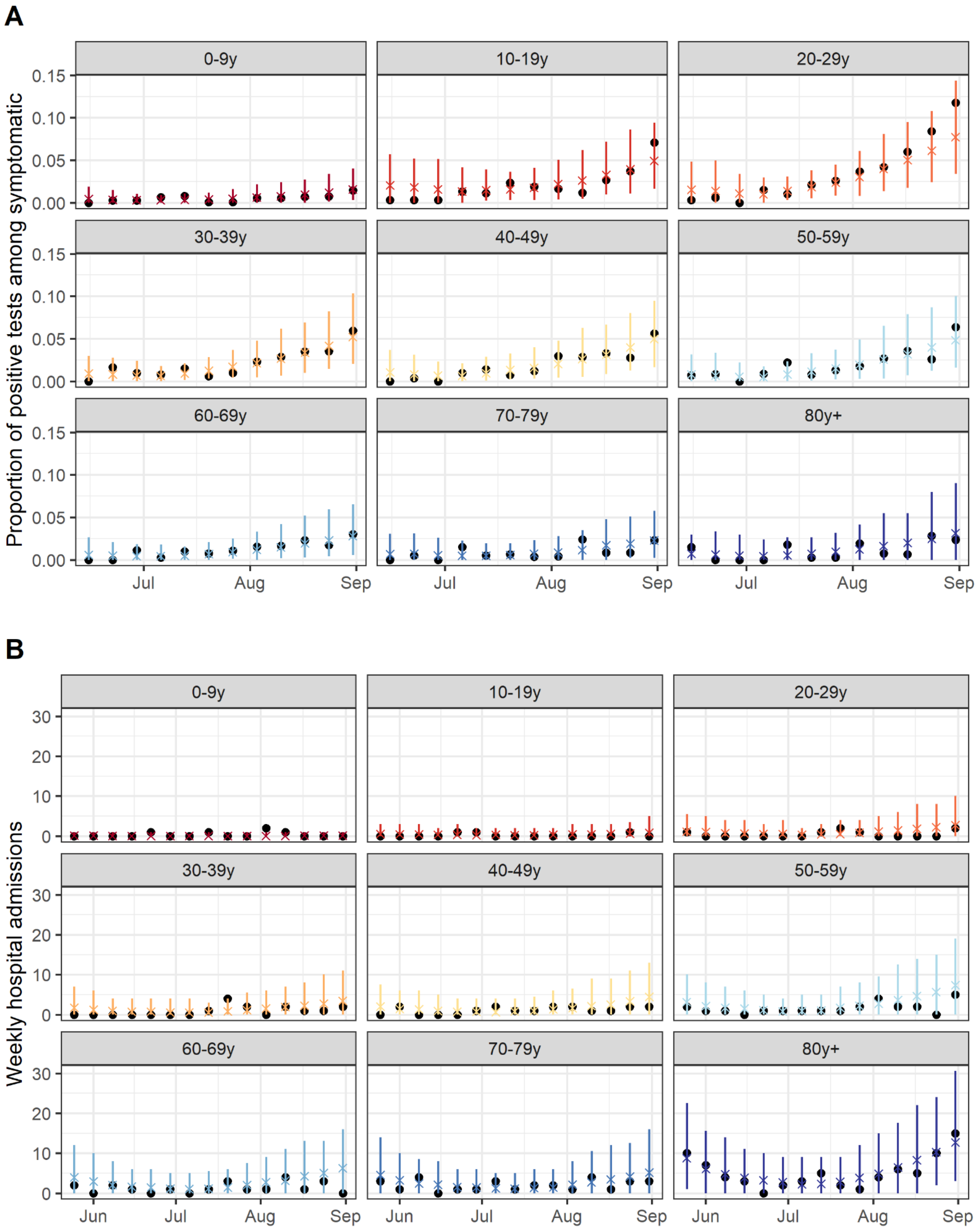

Supplementary Figure 16: Model predictions and observations in the Centre-Val de Loire region

A

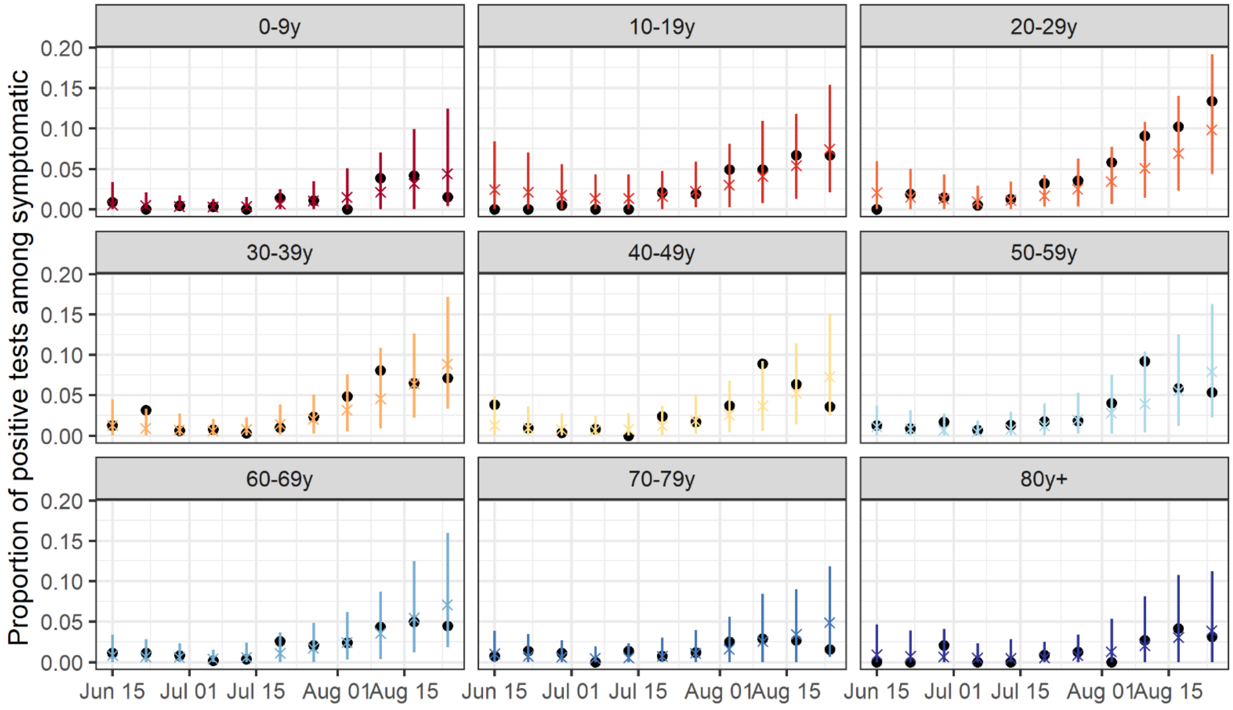

B

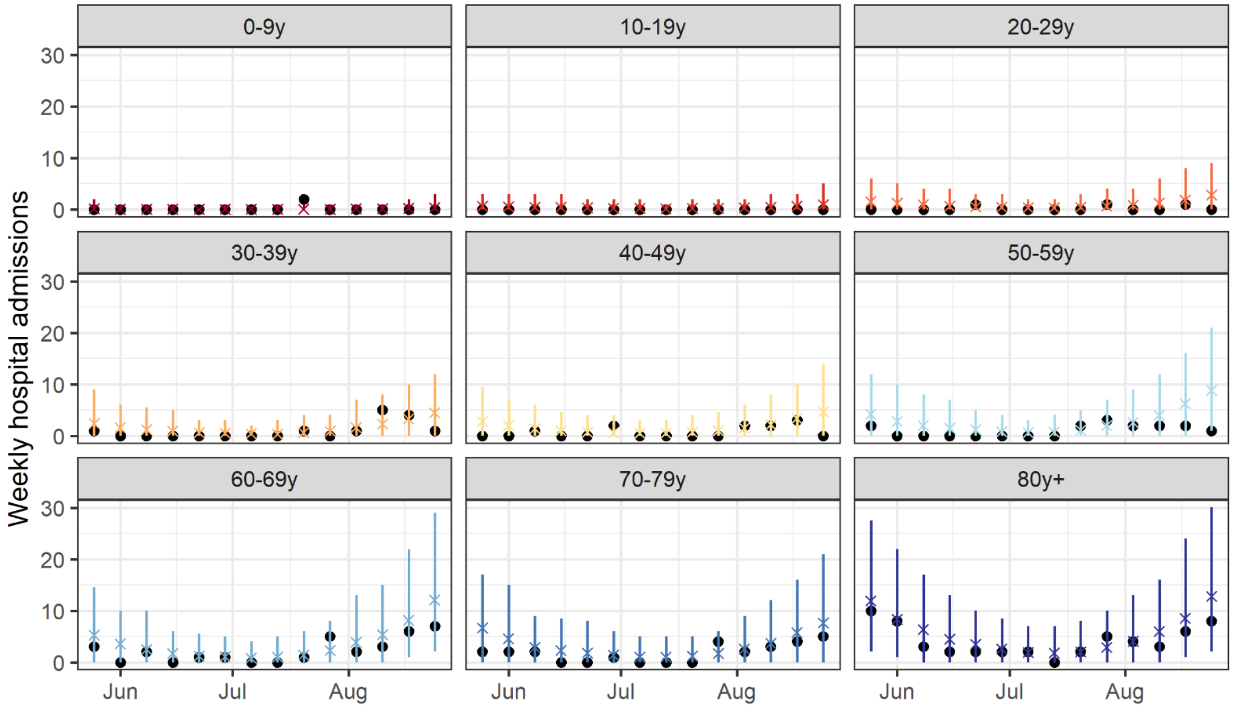

Supplementary Figure 17: Model predictions and observations in the Corse region

A

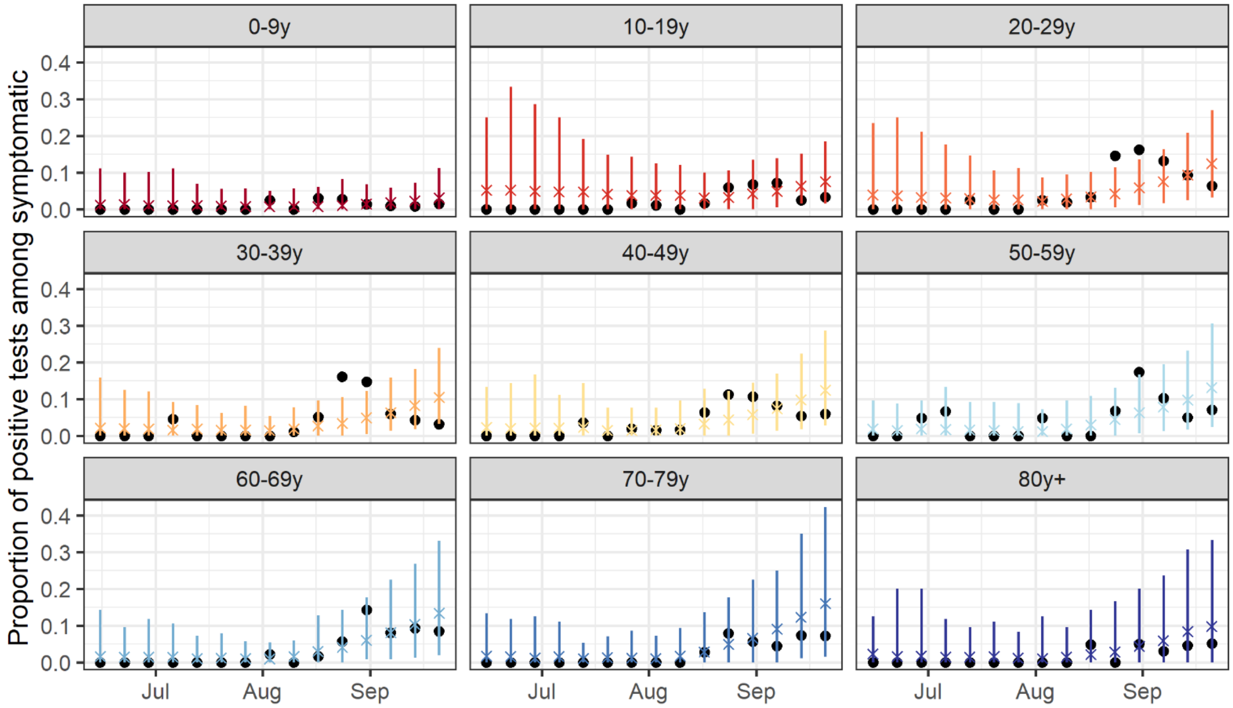

B

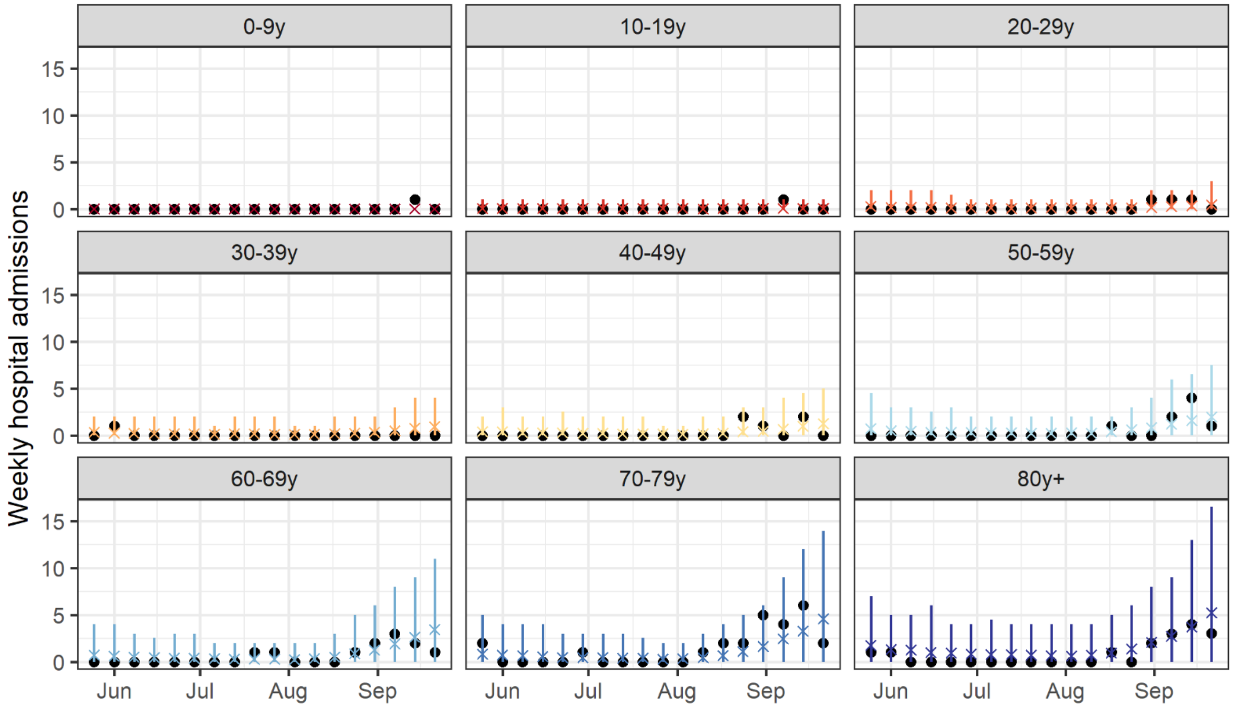

Supplementary Figure 18: Model predictions and observations in the Grand Est region

A

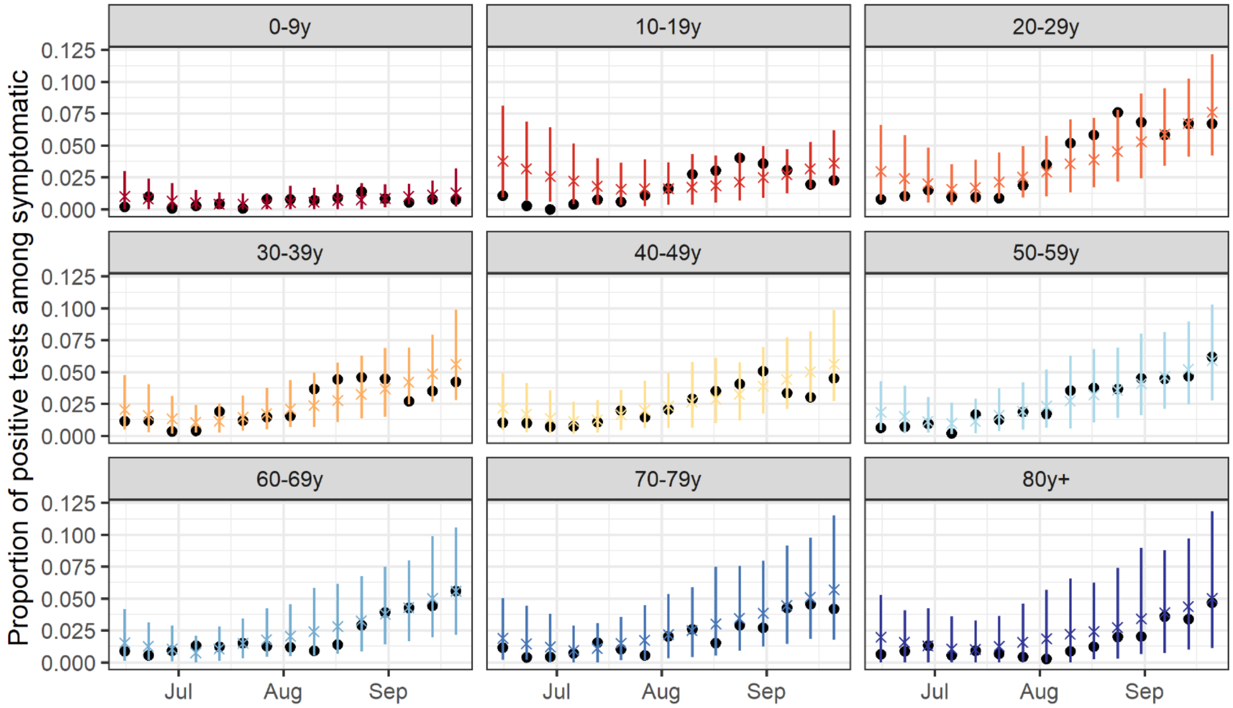

B

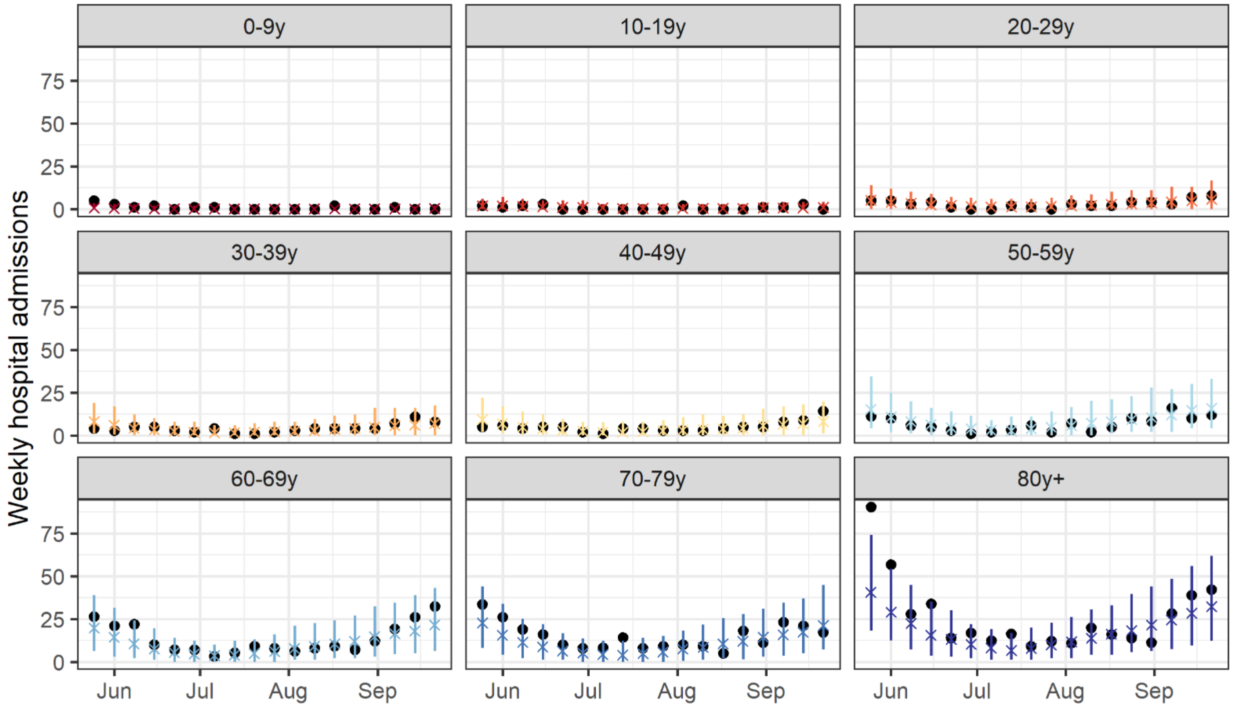

Supplementary Figure 19: Model predictions and observations in the Hauts-de-France region

A

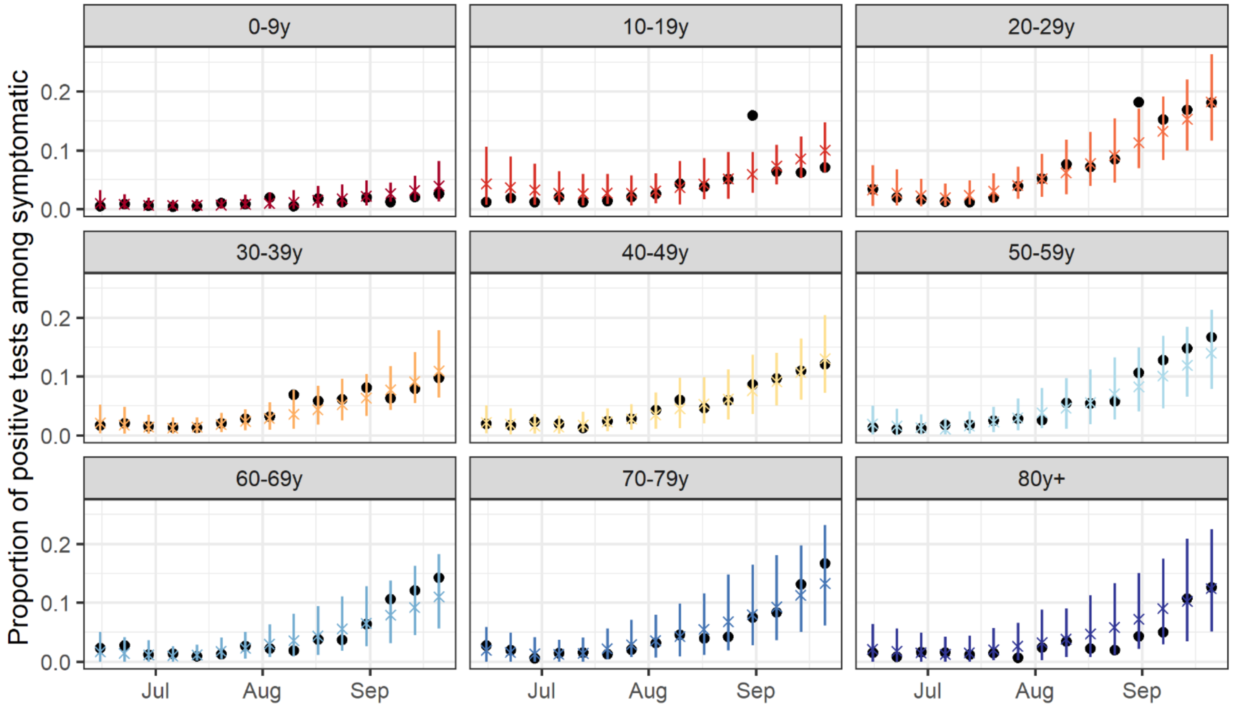

B

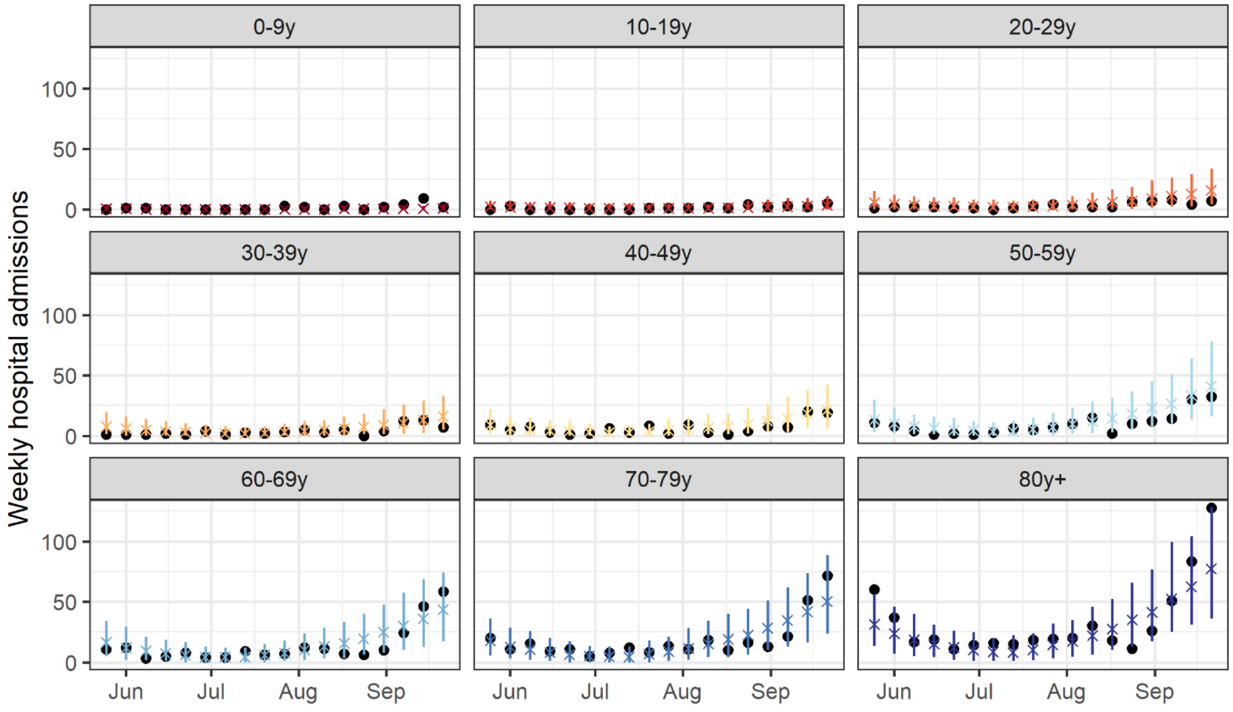

Supplementary Figure 20: Model predictions and observations in the Île-de-France region

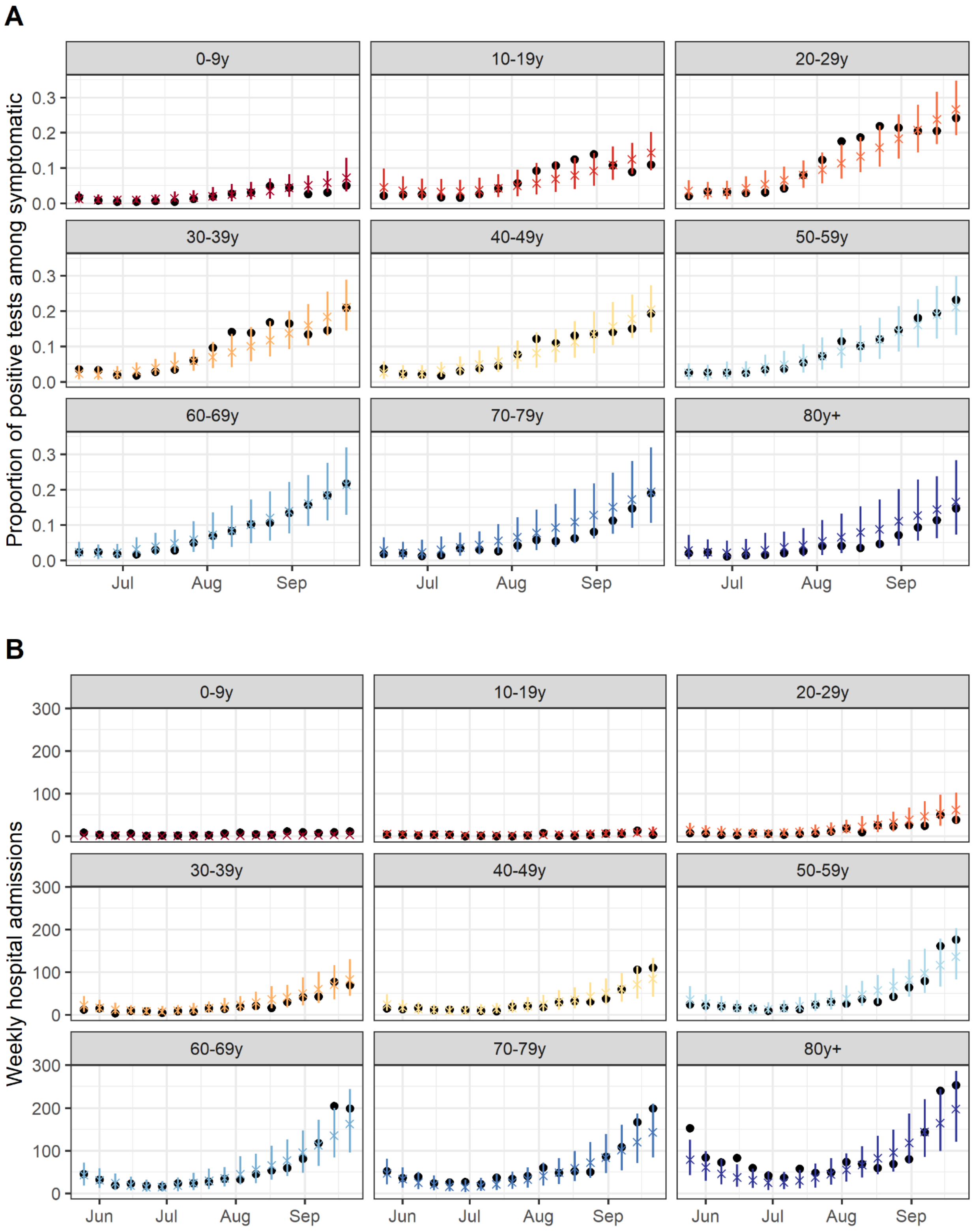

Supplementary Figure 21: Model predictions and observations in the Nouvelle-Aquitaine region

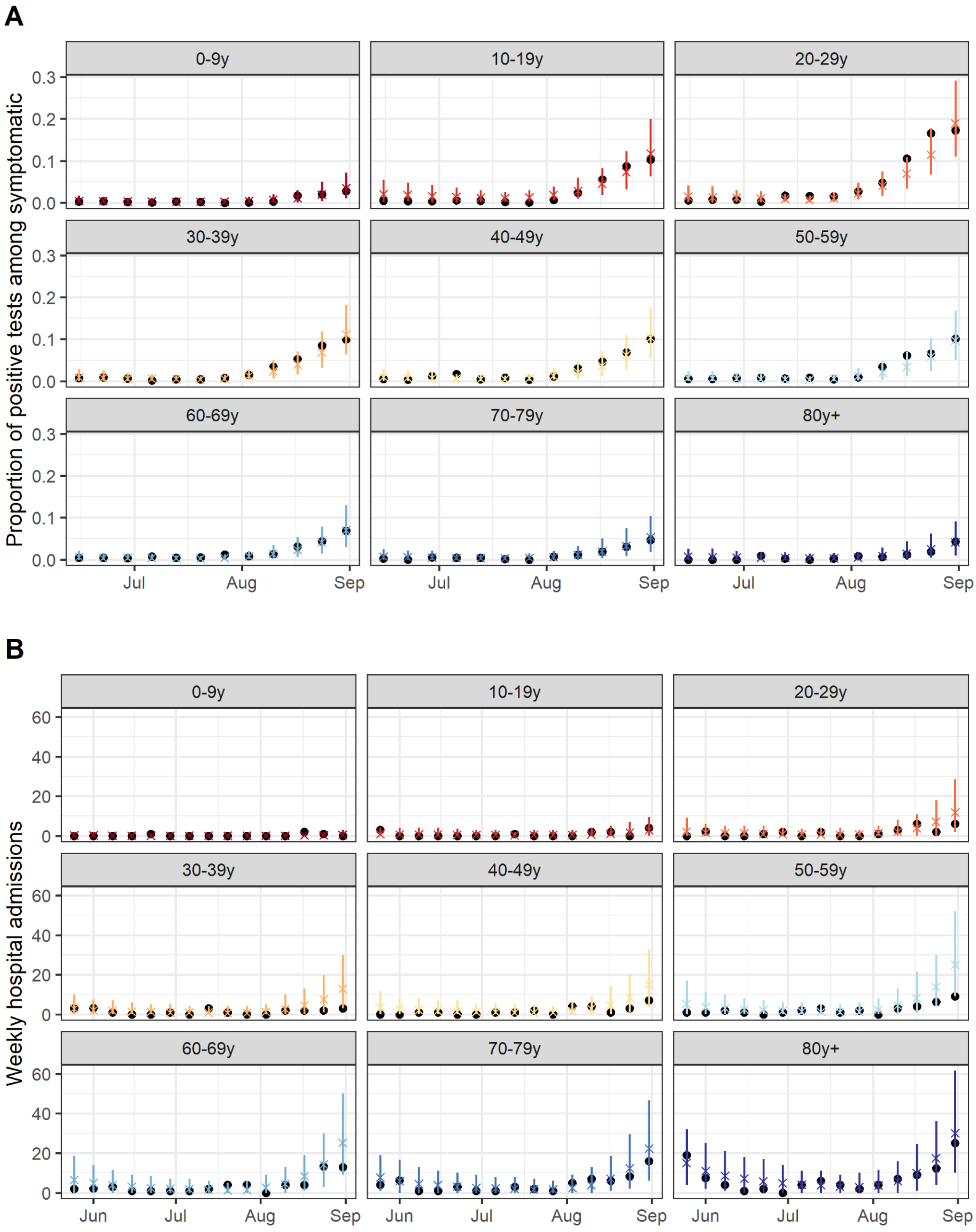

Supplementary Figure 22: Model predictions and observations in the Normandie region

A

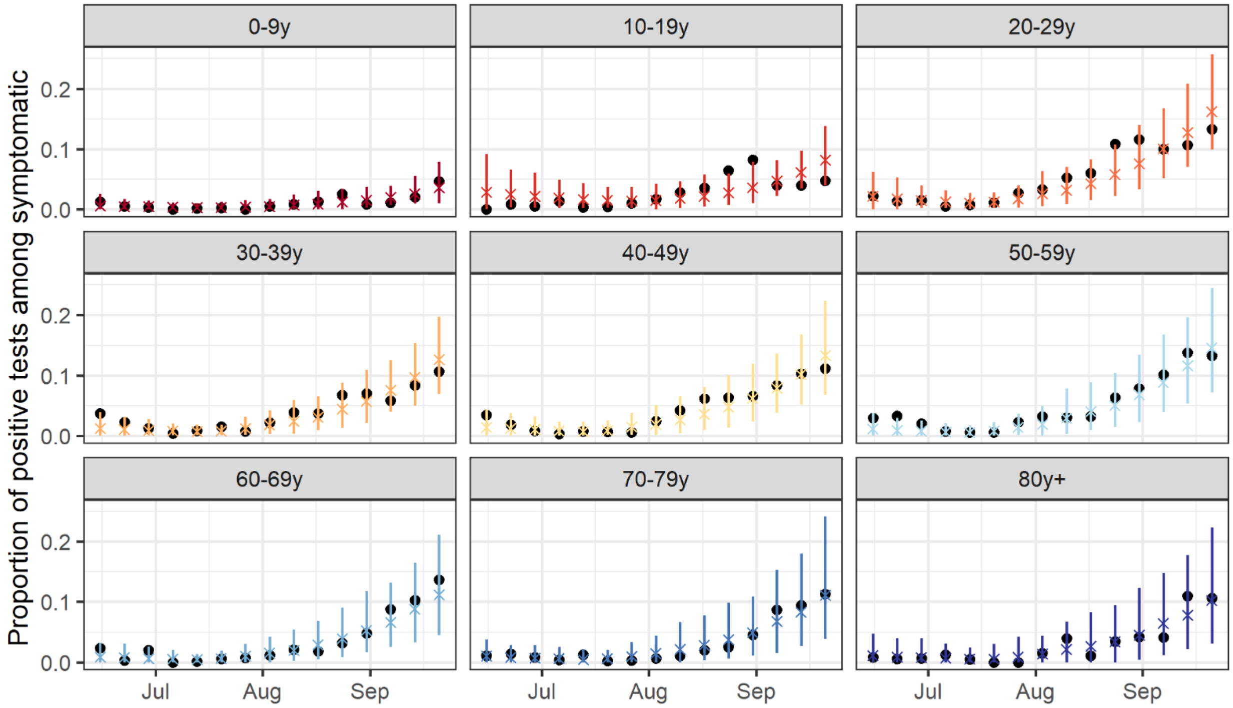

B

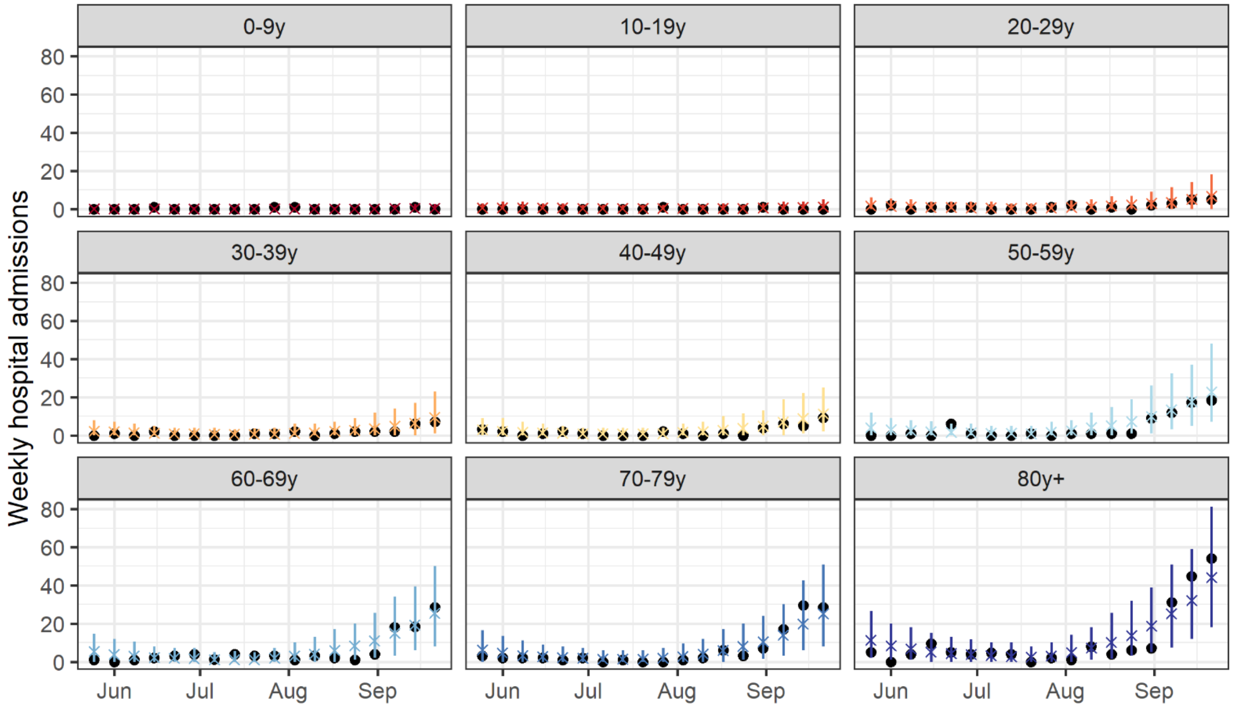

Supplementary Figure 23: Model predictions and observations in the Occitanie region

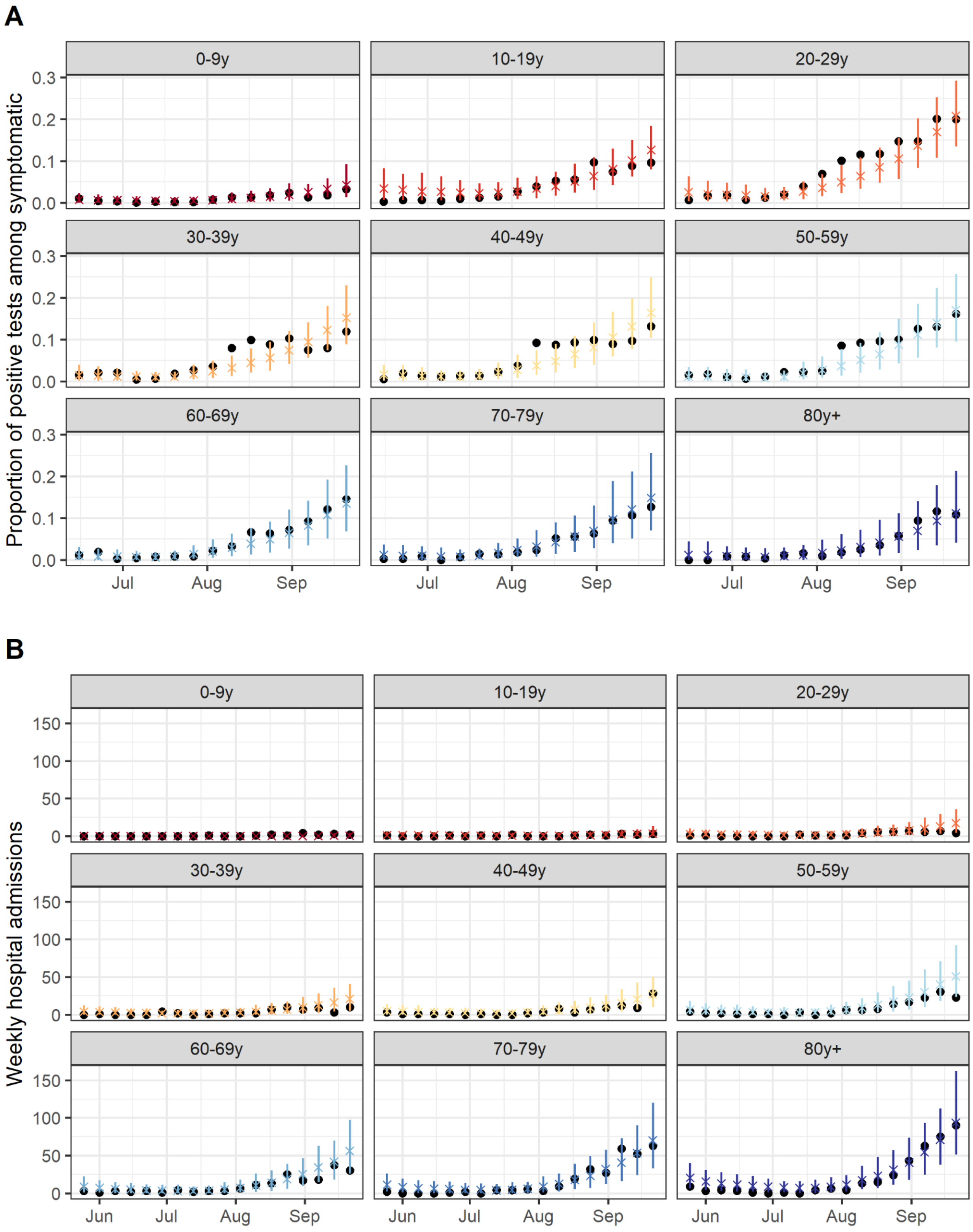

Supplementary Figure 24: Model predictions and observations in Provence-Alpes Côte d'Azur

A

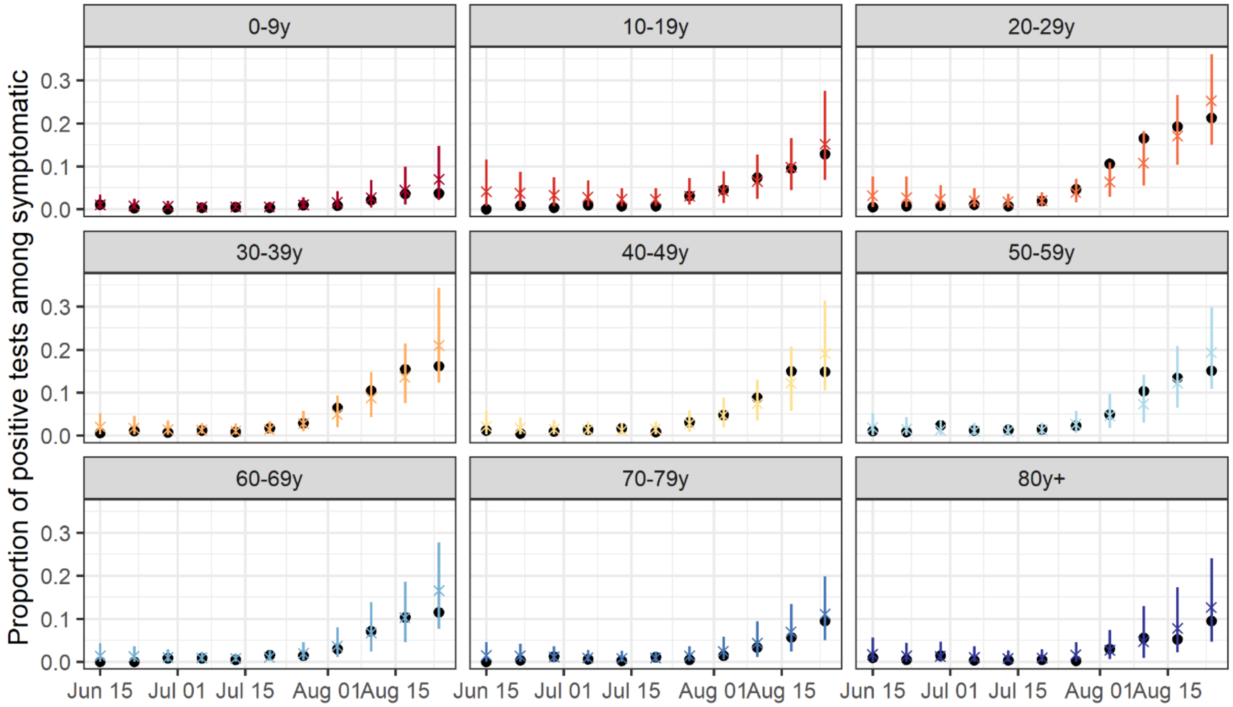

B

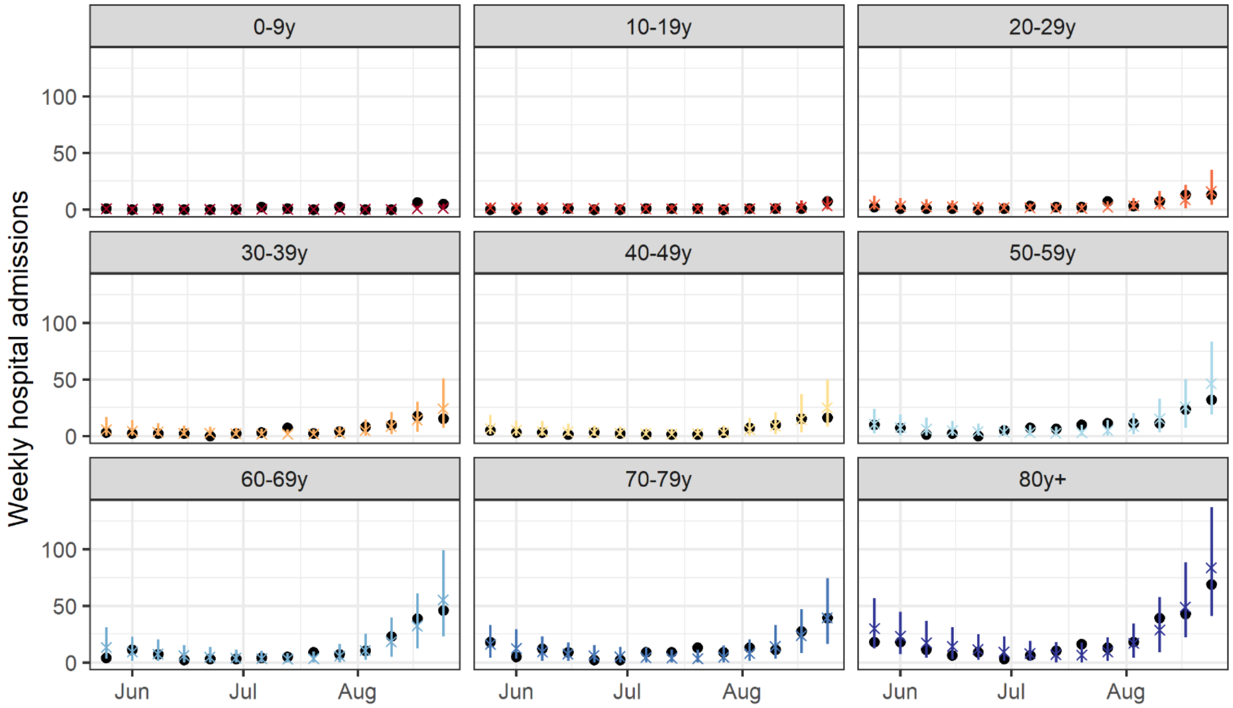

Supplementary Figure 25: Model predictions and observations in the Pays de la Loire region

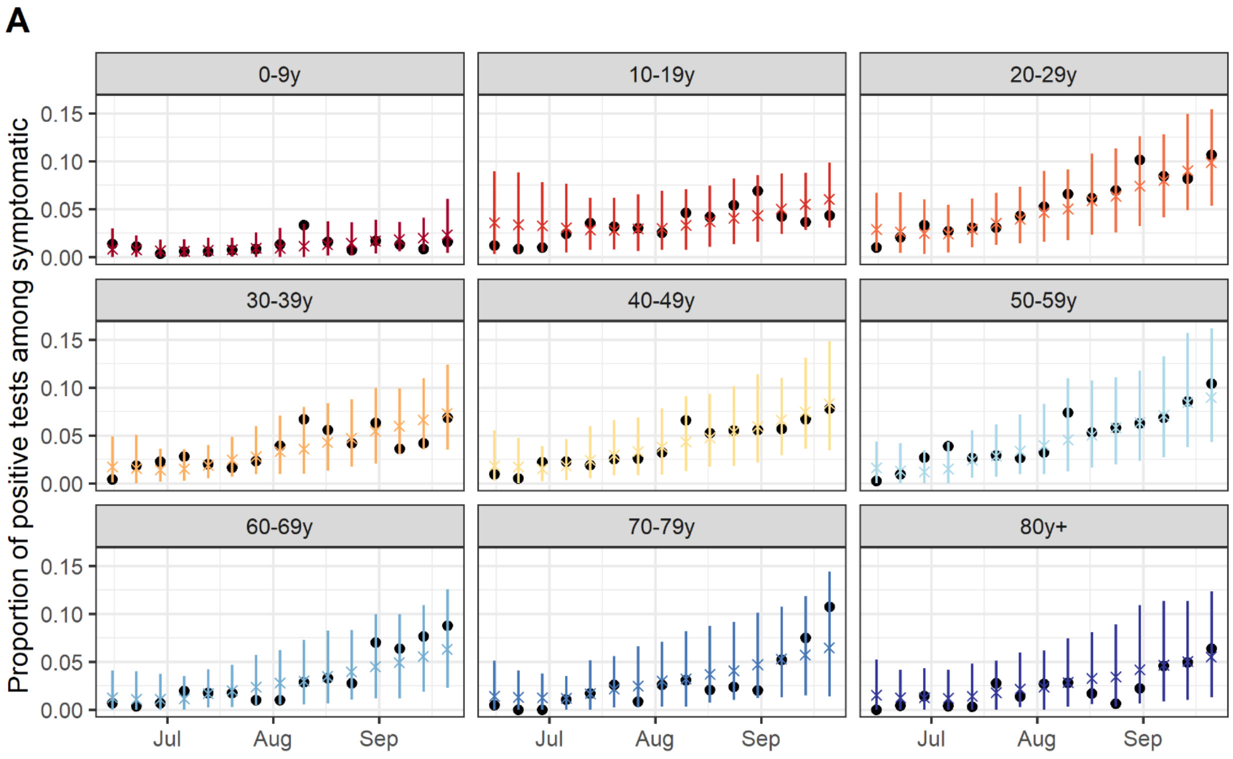

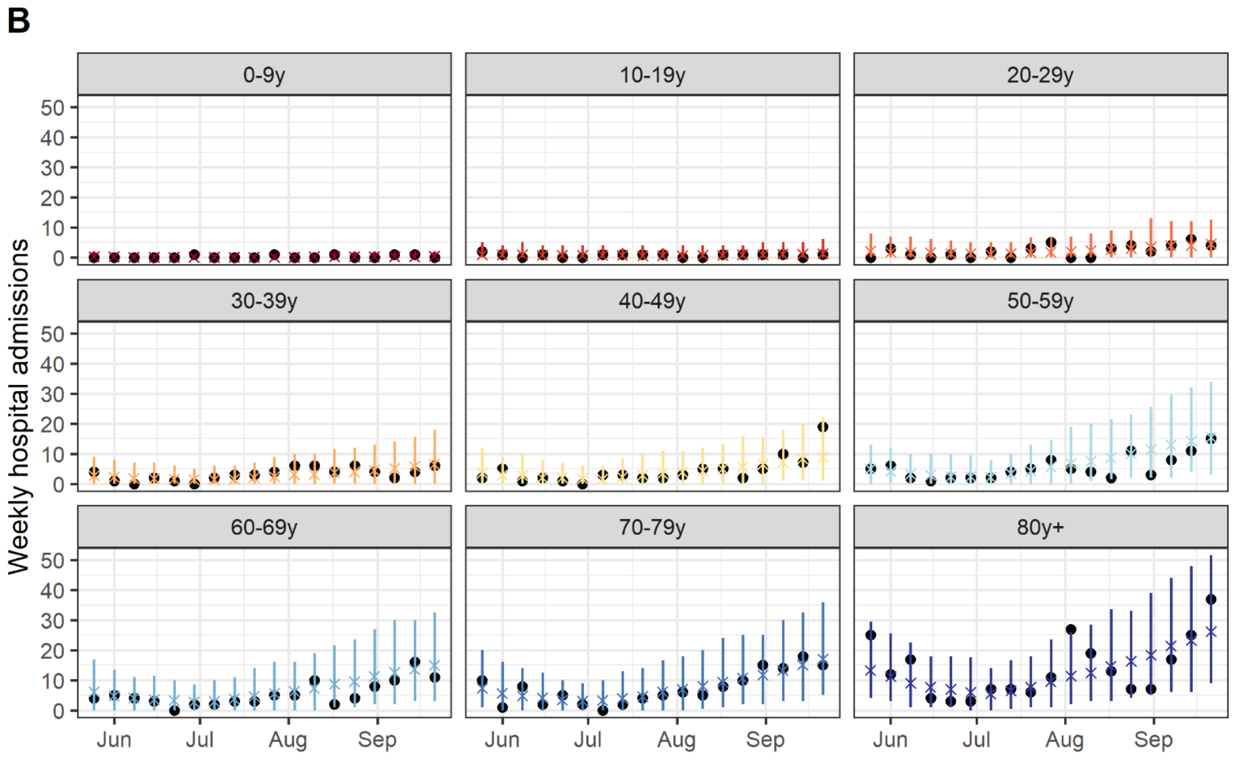

Supplementary Figure 26

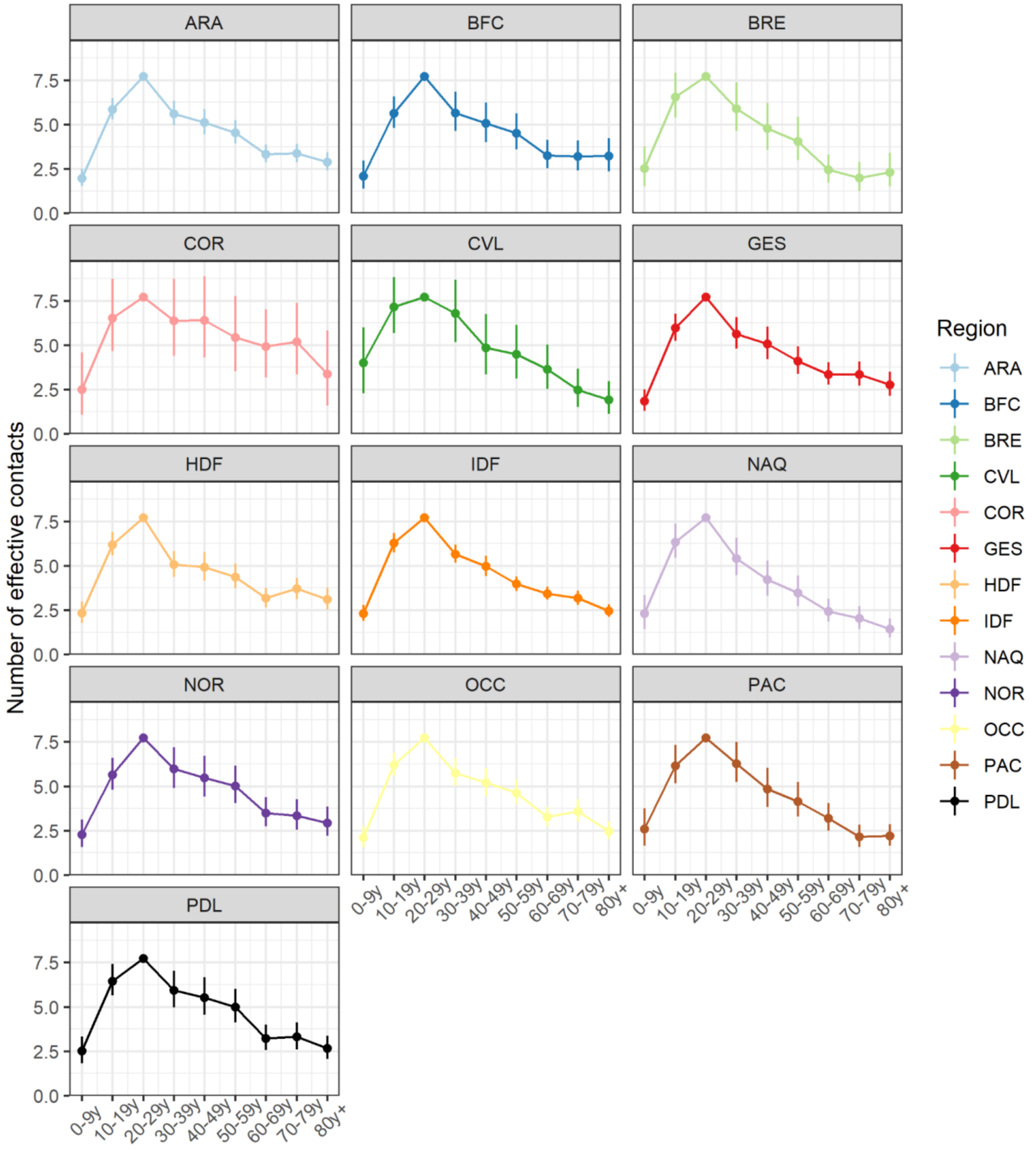

Supplementary Figure 26: Estimates of the number of contacts during the rebound period in the 13 regions of Metropolitan France. Predicted number of effective contacts in the different age groups during the rebound period. Regions' abbreviations are reported in the Supplementary Note 1. The vertical segments indicate 95% credible intervals obtained from the posterior distribution (chain of 100,000 iterations removing 5,000 iterations of burn-in).

Supplementary Figure 27

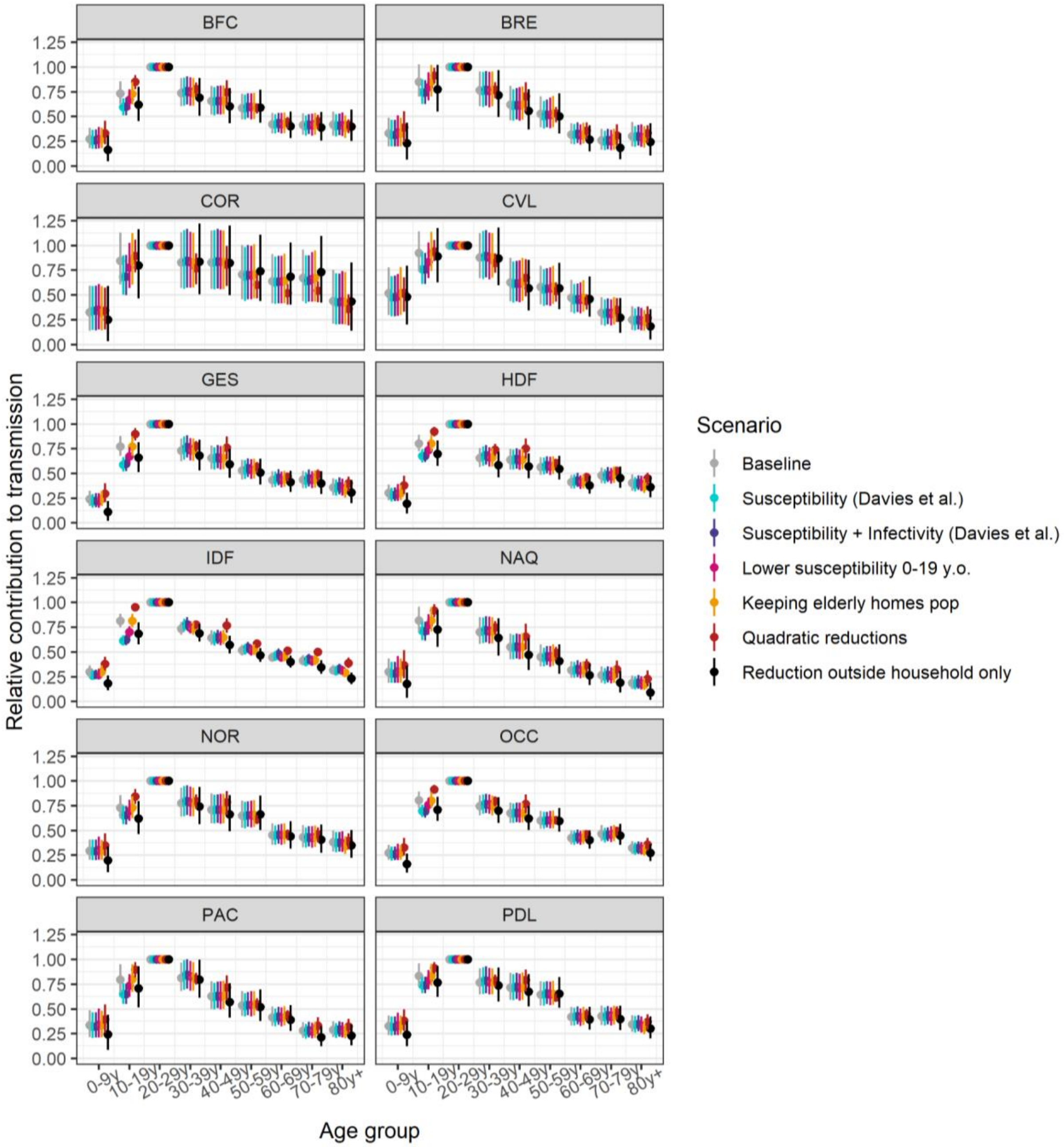

Supplementary Figure 27: Sensitivity analyses - Relative contribution to transmission of the different age groups in the different regions (except Auvergne-Rhône-Alpes). Different scenarios are explored: The scenarios explored are: *Susceptibility (Davies et al.)* - Using age-specific susceptibilities [13] ; *Susceptibility + Infectivity (Davies et al.)* - Using age-specific susceptibilities and infectivities [13]; *Lower susceptibility 0-19 y.o.* - 0-9 y.o. and 10-19 y.o. are respectively 50% and 25% less susceptible to SARS-CoV-2 infection than 20 y.o. and older ; *Keeping elderly homes pop* - Including the population of elderly homes in the study population ; *Quadratic reduction* - Considering quadratic reductions in contact patterns ; *Reduction outside household only* - Assuming contact patterns are only modified outside the household. The vertical segments indicate 95% credible intervals obtained from the posterior distribution (chain of 100,000 iterations removing 5,000 iterations of burn-in)

Supplementary Figure 28

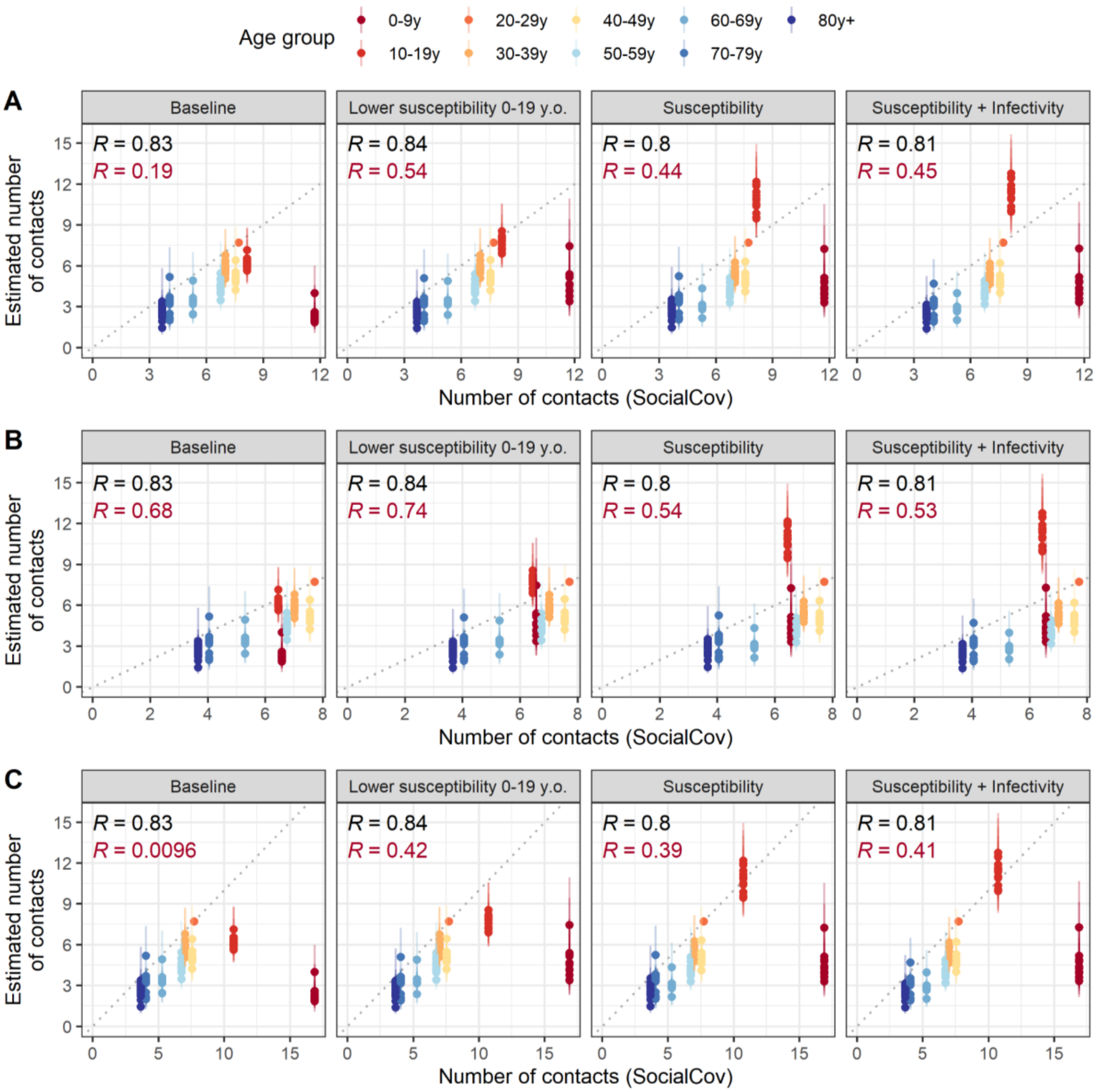

Supplementary Figure 28: Comparison between the estimated number of contacts and the number of contacts measured in the SocialCov survey. **(A)** Using the contact survey data for 0-19 y.o. between July 30th, 2020 and September 27th, 2020. **(B)** Using the contact survey data for 0-19 y.o. between July 30th, 2020 and September 1st, 2020. **(C)** Using the contact survey data for 0-19 y.o. between September 1st, 2020 and September 27th, 2020. Different scenarios are explored: *Susceptibility* - Using age-specific susceptibilities [13]; *Susceptibility* + *Infectivity* - Using age-specific susceptibilities and infectivities [13]; *Lower susceptibility 0-19 y.o.* - 0-9 y.o. and 10-19 y.o. are respectively 50% and 25% less susceptible to SARS-CoV-2 infection than 20 y.o. and older. Each point (with linerange) corresponds to the estimate for a given region with 95% credible interval obtained from the posterior distribution of parameters (MCMC chain of 100,000 iterations removing 5,000 iterations of burn-in). The upper values of R (black) correspond to the Pearson's correlation coefficient removing the 0-9 y.o. and 10-19 y.o. age groups. The lower values of R (red) correspond to the Pearson's correlation coefficient using the data from all age groups.

Supplementary Figure 29

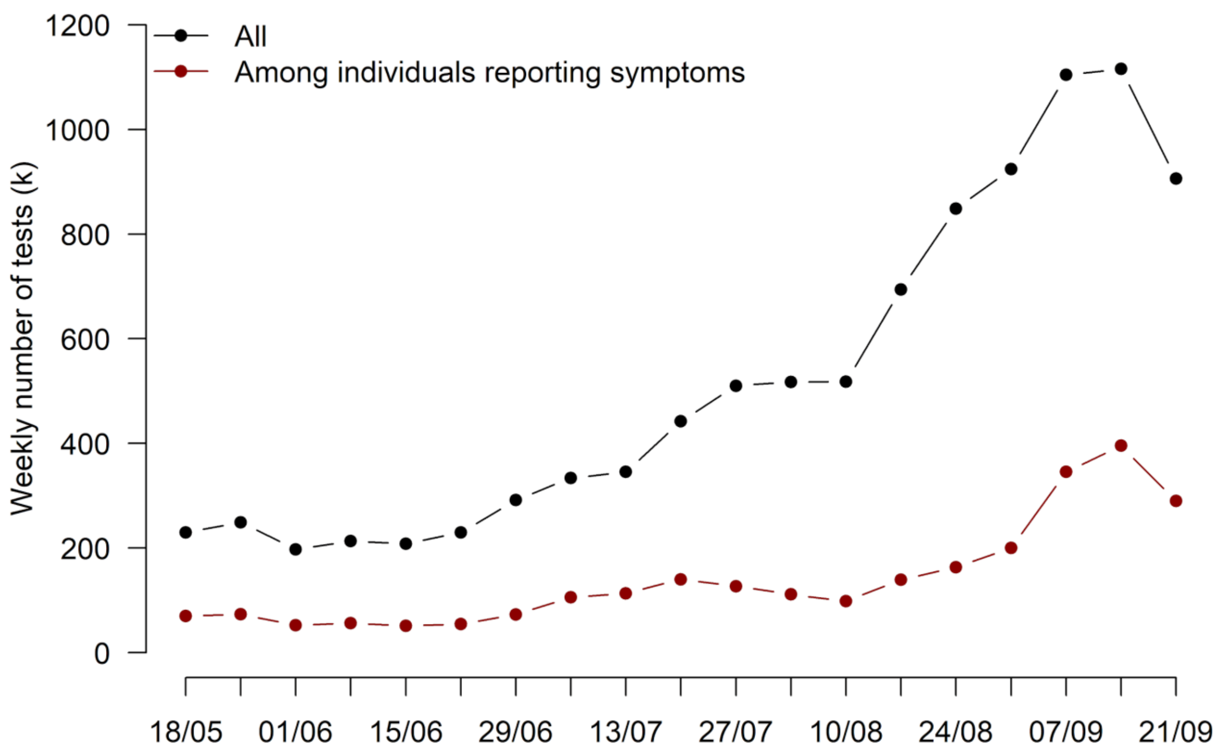

Supplementary Figure 29: Number of tests performed per week reported in the SIDEP surveillance system in metropolitan France.

Supplementary Figure 30

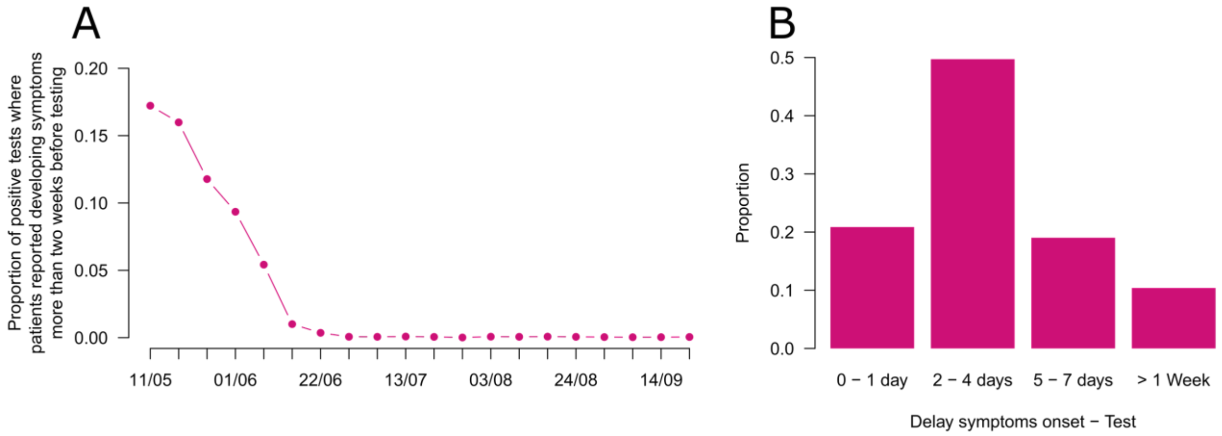

Supplementary Figure 30: Characteristics of the delay between onset of symptoms and test. (A) Proportion of positive tests in patients reporting a delay greater than two weeks between symptoms onset more and testing by week of nasopharyngeal swab. **(B)** Distribution of the delay between symptoms onset and test for the time period 15 June 2020 - 27 September 2020.

Supplementary Figure 31

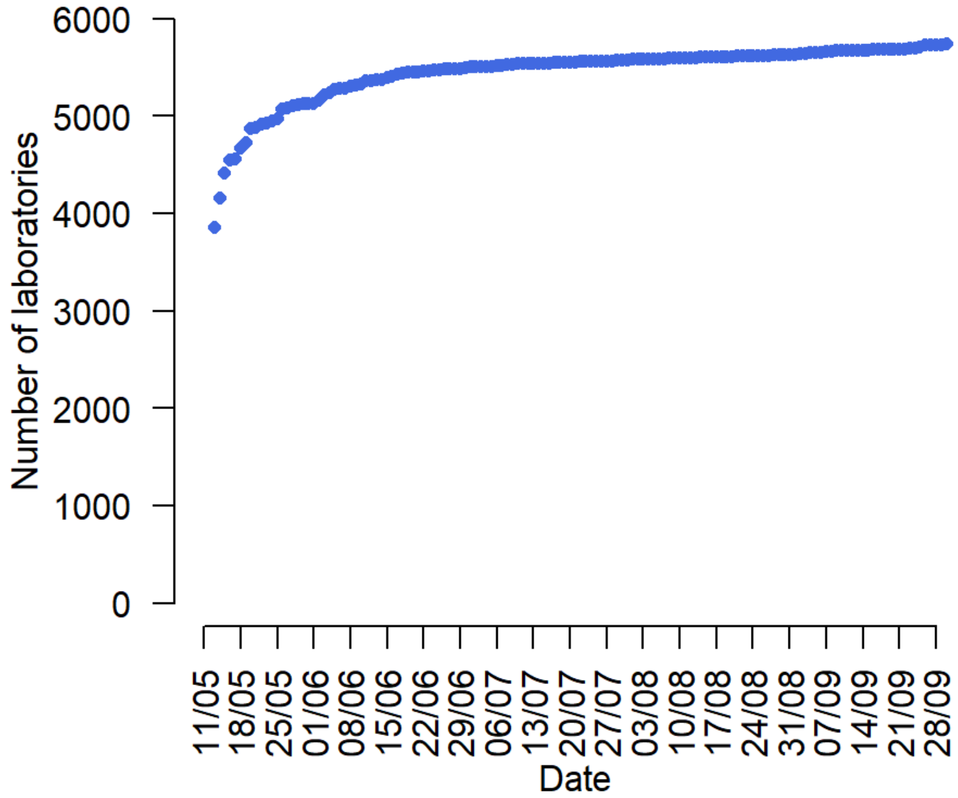

Supplementary Figure 31: Number of laboratories reporting in the SIDEP database through time.

Supplementary Table 1: Parameter 95% credible intervals

Parameters common to all the regions

Change in contact patterns during the post-lockdown period for individuals aged 0-9 y.o. ${\alpha_{0-9y}}^{postLock}$	0.51 (0.40 - 0.65)
Change in contact patterns during the post-lockdown period for individuals aged 10-19 y.o.${\alpha_{10-19y}}^{postLock}$	1.13 (0.90 - 1.37)
Change in contact patterns during the post-lockdown period for individuals aged 30-39 y.o.${\alpha_{30-39y}}^{postLock}$	0.81 (0.62 - 1.07)
Change in contact patterns during the post-lockdown period for individuals aged 40-49 y.o.${\alpha_{40-49y}}^{postLock}$	0.51 (0.41 - 0.62)
Change in contact patterns during the post-lockdown period for individuals aged 50-59 y.o.${\alpha_{50-59y}}^{postLock}$	0.62 (0.48 - 0.79)
Change in contact patterns during the post-lockdown period for individuals aged 60-69 y.o.${\alpha_{60-69y}}^{postLock}$	0.58 (0.46 - 0.71)
Change in contact patterns during the post-lockdown period for individuals aged 70-79 y.o.${\alpha_{70-79y}}^{postLock}$	0.64 (0.51 - 0.80)
Change in contact patterns during the post-lockdown period for individuals aged ≥80 y.o.${\alpha_{80y+}}^{postLock}$	0.77 (0.60 - 1.01)
Prevalence of non-COVID infections with COVID suggestive symptoms in the population π	0.0060 (0.0058 - 0.0063)
Overdispersion parameter associated with the contribution to the likelihood of age-stratified hospitalization data δ_2	0.64 (0.58 - 0.69)
Overdispersion parameter associated with the contribution to the likelihood of age-stratified test data δ_3	0.46 (0.44 - 0.49)

Region-specific transmission parameters

Region	Post-lockdown reproduction number $R_{postLock}$	Epidemic rebound reproduction number $R_{rebound}$
ARA	0.90 (0.88 - 0.93)	1.46 (1.44 - 1.49)
BFC	0.96 (0.93 - 0.99)	1.50 (1.46 - 1.55)
BRE	0.89 (0.86 - 0.93)	1.31 (1.25 - 1.36)

COR	1.03 (0.99 - 1.06)	1.40 (1.31 - 1.50)
CVL	0.86 (0.83 - 0.90)	1.54 (1.46 - 1.62)
GES	1.05 (1.02 - 1.08)	1.46 (1.43 - 1.49)
HDF	0.97 (0.95 - 1.00)	1.39 (1.36 - 1.42)
IDF	1.11 (1.08 - 1.15)	1.58 (1.56 - 1.60)
NAQ	0.90 (0.88 - 0.93)	1.72 (1.65 - 1.80)
NOR	0.91 (0.88 - 0.94)	1.40 (1.37 - 1.44)
OCC	0.96 (0.94 - 0.99)	1.38 (1.35 - 1.40)
PAC	0.96 (0.93 - 0.99)	1.81 (1.73 - 1.88)
PDL	0.98 (0.95 - 1.01)	1.20 (1.17 - 1.22)

Region-specific contact parameters ${\alpha_{Age}}^{rebound}$

	Age-group								
Region	0-9y	10-19y	20-29y	30-39y	40-49y	50-59y	60-69y	70-79y	80y+
ARA	0.30 (0.23 - 0.39)	0.61 (0.52 - 0.72)	1 (ref)	0.80 (0.62 - 1.04)	0.55 (0.46 - 0.67)	0.91 (0.63 - 1.39)	0.69 (0.54 - 0.89)	0.66 (0.52 - 0.85)	0.62 (0.50 - 0.80)
BFC	0.32 (0.21 - 0.47)	0.62 (0.48 - 0.78)	1 (ref)	0.80 (0.55 - 1.17)	0.56 (0.42 - 0.76)	1.01 (0.58 - 1.82)	0.77 (0.52 - 1.23)	0.67 (0.46 - 1.01)	0.91 (0.52 - 1.90)
BRE	0.39 (0.23 - 0.62)	0.74 (0.53 - 1.01)	1 (ref)	0.92 (0.54 - 1.51)	0.54 (0.36 - 0.79)	0.88 (0.46 - 1.81)	0.54 (0.34 - 0.85)	0.38 (0.23 - 0.60)	0.58 (0.32 - 1.26)
COR	0.44 (0.18 - 0.84)	0.78 (0.50 - 1.13)	1 (ref)	1.07 (0.59 - 1.82)	0.87 (0.51 - 1.42)	1.46 (0.63 - 3.12)	1.56 (0.74 - 2.99)	1.57 (0.77 - 2.91)	1.08 (0.39 - 3.07)
CVL	0.73	0.88	1 (ref)	1.17	0.58	1.08	1.04	0.54	0.48

	(0.38 - 1.30)	(0.61 - 1.20)		(0.66 - 1.97)	(0.37 - 0.89)	(0.52 - 2.22)	(0.56 - 1.94)	(0.30 - 0.88)	(0.26 - 0.80)
GES	0.27 (0.19 - 0.37)	0.61 (0.50 - 0.76)	1 (ref)	0.79 (0.57 - 1.12)	0.53 (0.42 - 0.68)	0.76 (0.51 - 1.23)	0.75 (0.54 - 1.11)	0.64 (0.47 - 0.90)	0.58 (0.42 - 0.85)
HDF	0.34 (0.25 - 0.44)	0.63 (0.53 - 0.76)	1 (ref)	0.64 (0.49 - 0.84)	0.50 (0.40 - 0.61)	0.80 (0.54 - 1.27)	0.63 (0.48 - 0.85)	0.71 (0.52 - 0.99)	0.61 (0.47 - 0.89)
IDF	0.33 (0.27 - 0.40)	0.61 (0.52 - 0.70)	1 (ref)	0.84 (0.68 - 1.03)	0.50 (0.43 - 0.58)	0.61 (0.50 - 0.75)	0.61 (0.51 - 0.73)	0.51 (0.43 - 0.61)	0.42 (0.36 - 0.50)
NAQ	0.32 (0.20 - 0.50)	0.67 (0.52 - 0.87)	1 (ref)	0.78 (0.51 - 1.21)	0.44 (0.32 - 0.59)	0.65 (0.40 - 1.15)	0.52 (0.34 - 0.79)	0.38 (0.25 - 0.56)	0.30 (0.19 - 0.45)
NOR	0.37 (0.25 - 0.52)	0.63 (0.49 - 0.79)	1 (ref)	0.88 (0.61 - 1.28)	0.63 (0.47 - 0.85)	1.28 (0.69 - 2.19)	0.86 (0.58 - 1.34)	0.70 (0.48 - 1.02)	0.70 (0.48 - 1.15)
OCC	0.32 (0.23 - 0.43)	0.67 (0.57 - 0.80)	1 (ref)	0.80 (0.62 - 1.07)	0.57 (0.47 - 0.70)	1.01 (0.67 - 1.58)	0.72 (0.56 - 0.96)	0.78 (0.58 - 1.10)	0.56 (0.44 - 0.71)
PAC	0.41 (0.25 - 0.62)	0.68 (0.51 - 0.89)	1 (ref)	1.05 (0.69 - 1.56)	0.55 (0.40 - 0.74)	0.86 (0.53 - 1.52)	0.77 (0.51 - 1.19)	0.43 (0.30 - 0.60)	0.52 (0.36 - 0.74)
PDL	0.41 (0.29 - 0.56)	0.75 (0.60 - 0.93)	1 (ref)	0.90 (0.63 - 1.29)	0.65 (0.50 - 0.85)	1.24 (0.70 - 2.07)	0.73 (0.53 - 1.06)	0.69 (0.49 - 0.99)	0.62 (0.45 - 0.85)

Supplementary Table 2: Mean daily number of contacts reported by participants of the SocialCov survey between 30 July 2020 and 27 September 2020.

**Age group**	**Mean daily number of contacts**	**95% bootstrap interval** (computed from 10,000 bootstrap samples)
0-9 y.o.	11.7	(10.0 - 13.5)
10-19 y.o.	8.1	(6.9 - 9.5)
20-29 y.o.	7.7	(6.9 - 8.7)
30-39 y.o.	7.0	(6.1 - 7.8)
40-49 y.o.	7.5	(6.8 - 8.4)
50-59 y.o.	6.7	(5.9 - 7.7)
60-69 y.o.	5.3	(4.4 - 6.4)
70-79 y.o.	4.1	(3.1 - 5.3)
≥80 y.o.	3.7	(1.3 - 6.4)

Supplementary Table 3: Dates used for a change in transmission levels in regions in Metropolitan France.

**Region**	**Date**
Auvergne-Rhône-Alpes	09/07/2020
Bourgogne-Franche-Comté	23/07/2020
Bretagne	06/07/2020
Centre-Val de Loire	09/07/2020
Corse	06/08/2020
Grand Est	09/07/2020
Hauts-de-France	09/07/2020
Île-de-France	25/06/2020
Nouvelle-Aquitaine	23/07/2020
Normandie	17/07/2020
Occitanie	17/07/2020
Provence Alpes Côte d’Azur	17/07/2020
Pays de la Loire	03/07/2020

Supplementary Table 4: Time windows used to calibrate the model in the different regions

Region	Time window
Auvergne-Rhône-Alpes	11/05/2020 - 27/09/2020
Bourgogne-Franche-Comté	11/05/2020 - 27/09/2020
Bretagne	11/05/2020 - 06/09/2020
Centre-Val de Loire	11/05/2020 - 31/08/2020
Corse	11/05/2020 - 27/09/2020
Grand Est	11/05/2020 - 27/09/2020
Hauts-de-France	11/05/2020 - 27/09/2020
Île-de-France	11/05/2020 - 27/09/2020
Nouvelle-Aquitaine	11/05/2020 - 06/09/2020
Normandie	11/05/2020 - 27/09/2020
Occitanie	11/05/2020 - 27/09/2020
Provence Alpes Côte d’Azur	11/05/2020 - 31/08/2020
Pays de la Loire	11/05/2020 - 27/09/2020

Supplementary Table 5: Probabilities of ICU admission and death given hospitalization used in forward simulations. These estimates are computed based on hospital admissions reported in the SI-VIC surveillance system in September and October 2020. We use the central estimates in the forward simulations. 95% confidence intervals were computed from 1,000,000 bootstrap samples.

Age-group	Probability of ICU admission given hospitalization	Probability of death given hospitalization
0-19 y.o.	12.7% (10.7% - 14.8%)	0.2% (0.0% - 0.5%)
20-29 y.o.	11.0% (9.4% - 12.7%)	0.3% (0.1% - 0.6%)
30-39 y.o.	16.1% (14.6% - 17.6%)	1.1% (0.7% - 1.5%)
40-49 y.o.	20.8% (19.5% - 22.2%)	2.3% (1.8% - 2.7%)
50-59 y.o.	25.6% (24.4% - 26.7%)	4.5% (4.0% - 5.0%)
60-69 y.o.	32.1% (31.2% - 33.0%)	11.0% (10.4% - 11.6%)
70-79 y.o.	28.0% (27.2% - 28.8%)	18.6% (17.9% - 19.3%)
≥80 y.o.	8.5% (8.1% - 8.9%)	30.6% (30.0% - 31.1%)

Supplementary Table 6: Percentage of hospital deaths arising among patients hospitalized in ICUs. These estimates are computed based on hospital admissions reported in the SI-VIC surveillance system in September and October 2020. We use the central estimates in the forward simulations (to compute quality adjusted life years). 95% confidence intervals were computed from 1,000,000 bootstrap samples.

Age-group	Proportion of deaths occurring in ICUs
0-19 y.o.	50% (0% - 100%)
20-29 y.o.	75% (25% - 100%)
30-39 y.o.	64% (44% - 84%)
40-49 y.o.	61% (51% - 71%)
50-59 y.o.	61% (55% - 66%)
60-69 y.o.	67% (64% - 70%)
70-79 y.o.	55% (52% - 57%)
≥80 y.o.	14% (13% - 15%)

Supplementary Table 7: Weights used to compute the number of life years lost and the number of quality adjusted life years lost.

Age group	Weights for the computation of the number of life years lost	Weights for the computation of the number of quality adjusted life years lost
0-9 y.o.	78.4 years	66.6 years
10-19 y.o.	65.5 years	56.7 years
20-29 y.o.	58.7 years	47.2 years
30-39 y.o.	49.0 years	38.5 years
40-49 y.o.	39.4 years	30.3 years
50-59 y.o.	30.4 years	22.9 years
60-69 y.o.	22.1 years	16.2 years
70-79 y.o.	14.4 years	10.3 years
≥80 y.o.	6.9 years	4.9 years

References

1. Visseaux, B. *et al.* Prevalence of respiratory viruses among adults, by season, age, respiratory tract region and type of medical unit in Paris, France, from 2011 to 2016. *PLoS One* **12**, e0180888 (2017).

2. Diekmann, O., Heesterbeek, J. A. & Metz, J. A. On the definition and the computation of the basic reproduction ratio R0 in models for infectious diseases in heterogeneous populations. *J. Math. Biol.* **28**, 365–382 (1990).

3. Salje, H. *et al.* Estimating the burden of SARS-CoV-2 in France. *Science* **369**, 208–211 (2020).

4. Lefrancq, N. *et al.* Evolution of outcomes for patients hospitalised during the first 9 months of the SARS-CoV-2 pandemic in France: A retrospective national surveillance data analysis. *The Lancet Regional Health - Europe* vol. 5 100087 (2021).

5. Insee − Institut national de la statistique et des études économiques. https://www.insee.fr/.

6. Chevalier, J. & de Pouvourville, G. Valuing EQ-5D using time trade-off in France. *Eur. J. Health Econ.* **14**, 57–66 (2013).

7. Sandmann, F. *et al.* The potential health and economic value of SARS-CoV-2 vaccination alongside physical distancing in the UK: transmission model-based future scenario analysis and economic evaluation. doi:10.1101/2020.09.24.20200857.

8. van Hoek, A. J., Underwood, A., Jit, M., Miller, E. & Edmunds, W. J. The impact of pandemic influenza H1N1 on health-related quality of life: a prospective population-based study. *PLoS One* **6**, e17030 (2011).

9. van Hoek, A. J., Underwood, A., Jit, M., Miller, E. & Edmunds, W. J. The impact of pandemic influenza H1N1 on health-related quality of life: a prospective population-based study. *PLoS One* **6**, e17030 (2011).

10. Baguelin, M., Camacho, A., Flasche, S. & John Edmunds, W. Extending the elderly- and risk-group programme of vaccination against seasonal influenza in England and Wales: a

cost-effectiveness study. *BMC Medicine* vol. 13 (2015).

11. Cuthbertson, B. H., Roughton, S., Jenkinson, D., Maclennan, G. & Vale, L. Quality of life in the five years after intensive care: a cohort study. *Crit. Care* **14**, R6 (2010).
12. Griffiths, J. *et al.* An exploration of social and economic outcome and associated health-related quality of life after critical illness in general intensive care unit survivors: a 12-month follow-up study. *Critical Care* vol. 17 R100 (2013).
13. Davies, N. G. *et al.* Age-dependent effects in the transmission and control of COVID-19 epidemics. *Nature Medicine* vol. 26 1205–1211 (2020).
14. Li, R. *et al.* Substantial undocumented infection facilitates the rapid dissemination of novel coronavirus (SARS-CoV-2). *Science* **368**, 489–493 (2020).
15. Viner, R. M. *et al.* Susceptibility to SARS-CoV-2 Infection Among Children and Adolescents Compared With Adults: A Systematic Review and Meta-analysis. *JAMA Pediatr.* **175**, 143–156 (2021).
16. O'Driscoll, M. *et al.* Age-specific mortality and immunity patterns of SARS-CoV-2. *Nature* **590**, 140–145 (2021).
17. Zhang, J. *et al.* Changes in contact patterns shape the dynamics of the COVID-19 outbreak in China. *Science* **368**, 1481–1486 (2020).
18. Béraud, G. *et al.* The French Connection: The First Large Population-Based Contact Survey in France Relevant for the Spread of Infectious Diseases. *PLoS One* **10**, e0133203 (2015).
19. Bosetti, P. *et al.* Lockdown impact on age-specific contact patterns and behaviours in France. (2020) doi:10.1101/2020.10.07.20205104.
